# Maternal cholesterol deficiency predisposes congenital heart defects risk

**DOI:** 10.1038/s41392-025-02463-w

**Published:** 2025-11-12

**Authors:** Yayun Gu, Jimiao Gao, Hong Lv, Yan Zhou, Tao Jiang, Jia Guo, Wanting Ma, Yiwei Cheng, Xia Chi, Qi Xi, Kan Ye, Jiangbo Du, Jiong Li, Cheng Wang, Juncheng Dai, Hongxia Ma, Guangfu Jin, Yuan Lin, Hongbing Shen, Zhibin Hu

**Affiliations:** 1 State Key Laboratory of Reproductive Medicine and offspring health, Center for Global Health, School of Public Health, Nanjing Medical University, Nanjing, Jiangsu 211100 China; 2 State Key Laboratory of Reproductive Medicine and Offspring Health (Suzhou Centre), The Affiliated Suzhou Hospital of Nanjing Medical University, Suzhou Municipal Hospital, Gusu School, Nanjing Medical University, Suzhou, 215002 China; 3 Jiangsu Province Collaborative Innovation Center for Cardiovascular Disease Translational Medicine, Nanjing, Jiangsu 210000 China; 4 Department of Child Health Care, Women’s Hospital of Nanjing Medical University, Nanjing Maternity and Child Health Care Hospital, Nanjing, Jiangsu 210000 China; 5 Department of Obstetrics, The Affiliated Suzhou Hospital of Nanjing Medical University, Suzhou Municipal Hospital, Gusu School, Nanjing Medical University, Suzhou, 215002 Jiangsu China; 6 Department of Child Health Care, The Affiliated Suzhou Hospital of Nanjing Medical University, Suzhou Municipal Hospital, Gusu School, Nanjing Medical University, Suzhou, 215002 Jiangsu China

**Keywords:** Developmental biology, Cardiology

## Abstract

The relationship between maternal cholesterol deficiency and the risk of congenital heart defects (CHDs) in offspring is not fully understood. In a birth cohort study of 5041 family trios, we found that low maternal cholesterol levels were significantly associated with an increased risk of CHD, with RRs of 1.52 in the second trimester and 1.73 in the third trimester. To further investigate this link, we treated pregnant mice with cholesterol-lowering agents, namely, ezetimibe or atorvastatin. Both treatments led to a significant increase in the incidence of CHD in offspring. To identify a pathogenic variant that could provide genetic evidence linking cholesterol synthesis to CHD occurrence and serve as a target for constructing a genetic mouse model, we performed whole-genome sequencing (WGS) on 103 CHD cases from the birth cohort. We identified a recurrent functional variant in the *CYP51A1* gene (c.1147 A > G, p.Ile383Val). We then developed a *Cyp51*^*I383V*^ knock-in mouse model. This variant disrupted cholesterol synthesis, resulting in CHD through impaired hedgehog (Hh) signaling. Most intriguingly, maternal dietary intervention to increase cholesterol intake effectively reduced the risk of CHD in *Cyp51*^*I383V*^ mutant offspring. Our study suggests that low maternal cholesterol during pregnancy increases the risk of CHD in offspring by inhibiting Hh signaling and that maternal cholesterol supplementation during pregnancy may reduce the occurrence of CHD.

## Introduction

Congenital heart disease (CHD), the most common birth defect and the leading cause of perinatal and infant mortality worldwide,^1,2^ is a broad spectrum of structural defects affecting the heart and large vessels formed during fetal development. While a subset of CHDs can be attributed to well-characterized genetic variants, a large fraction of CHDs remains unexplained, suggesting a complex interplay between genetic susceptibilities and environmental factors during gestation. In recent years, the role of the intrauterine metabolic environment in influencing embryonic development has gained significant attention. Metabolites, including lipids,^3^ amino acids,^4^ nucleotides,^5^ are no longer seen solely as energy sources but as critical signaling molecules that regulate key developmental processes such as cell proliferation, differentiation, and lineage specification.^6^ Among these metabolites, cholesterol is a key component and a structural component of cell membranes that governs membrane integrity and fluidity, a precursor for steroid hormones and bile acids, and a critical modulator of developmental signaling pathways, most notably the Hedgehog (Hh) cascade.^7^ A balanced metabolism of cholesterol ensures the structural integrity, thickness, permeability, and fluidity of cell membranes,^8^ all of which are vital for normal development. Covalent cholesterol modification of Hh and Smoothened (SMO) proteins has been shown to be indispensable for their signaling activity,^9–11^ with reduced activity linked to a spectrum of developmental anomalies, including holoprosencephaly, brachydactyly, and abnormalities in cardiogenesis.^12–14^ Furthermore, noncovalent cholesterol-protein interactions have been detected for hundreds of proteins, although their developmental significance remains poorly understood.^15^ Collectively, these observations suggest that disruption of cholesterol homeostasis has clear potential to perturb fundamental developmental programs, including cardiogenesis.

The precise relationships between maternal/fetal cholesterol levels and CHD risk remain incompletely unresolved. Resolving these relationships is clinically important. A retrospective cohort study investigating pregnant women exposed to statin therapy (inhibitors of HMG-CoA reductase, the rate-limiting enzyme in cholesterol biosynthesis) during their first trimester revealed an increased risk of fetal congenital cardiac anomalies.^16^ However, conflicting findings have also been reported,^17^ suggesting that the association between cholesterol deficiency and cardiac defects remains largely unclear.^18^ This inconsistency highlights that the effect of statin therapy on fetal cardiogenesis might be more complex and potentially dependent on factors such as dosage, timing, and maternal cholesterol levels. Such variability underscores the necessity for a more comprehensive understanding of how maternal cholesterol dynamics influence fetal cardiogenesis and the underlying mechanisms responsible for CHD risk. A better grasp of how maternal and embryonic cholesterol dynamics interact to influence CHD risk could therefore inform prenatal screening, risk stratification, and potentially nutritional or pharmacological interventions aimed at reducing disease burden.

To address these questions, we employed an integrated research strategy combining human epidemiology, genetic studies, pharmacologic perturbation in mice, and mechanistic experimentation. First, we examined maternal serum cholesterol levels and offspring cardiac outcomes in a prospective birth cohort of 5041 family trios, measuring cholesterol during routine antenatal visits in the second and third trimesters and assessing fetal/infant cardiac defects via color sonography. Second, we modeled maternal cholesterol reduction in mice: ICR females received either the intestinal cholesterol absorption inhibitor ezetimibe or the HMG-CoA reductase inhibitor atorvastatin, followed by embryo transfer and assessment of embryonic cardiac cholesterol and postnatal cardiac phenotypes. Third, we investigated genetic contributors by characterizing a missense variant in *CYP51A1*, a key lanosterol demethylase in the cholesterol biosynthesis pathway, and pursued mechanistic studies examining in situ cholesterol synthesis in embryonic hearts, Hh signaling activity, ciliogenesis, and the expression of cardiac transcription factors such as GATA4. Finally, we tested whether maternal high-cholesterol diets could reverse genetically or pharmacologically induced cardiac defects, providing potential insights into therapeutic strategies for managing CHD risk.

This study represents one of the first attempts to systematically integrate population-based cohort data, genomic data, and experimental models to explore the role of maternal and embryonic cholesterol dynamics in CHD pathogenesis. We identified an association between low maternal cholesterol and increased CHD risk in a large birth cohort. In line with these human findings, our mouse model studies demonstrated that pharmacological reduction of maternal cholesterol during pregnancy resulted in similar cardiac defects in offspring, further supporting the hypothesis that cholesterol deficiency can lead to congenital heart anomalies. We further pinpointed a missense variant in the *CYP51A1* gene that disrupts embryonic cholesterol synthesis, leading to impaired Hedgehog signaling, reduced GATA4 expression, defective ciliogenesis, and consequent heart malformations, highlighting the importance of cholesterol in the early stages of cardiac development. Importantly, supplementation with a high-cholesterol maternal diet significantly reduced the incidence of CHD in mouse offspring, revealing a reversible pathogenic mechanism and providing key insights into potential preventive strategies for human CHD. This work is novel in that it provides convergent evidence from both human and animal studies, revealing the crucial role of cholesterol homeostasis in cardiogenesis and offering new insights into potential strategies for preventing CHD. By bridging basic science with clinical implications, our study not only advances our understanding of the molecular underpinnings of CHDs but also lays the groundwork for future research focused on cholesterol-based interventions in maternal-fetal medicine.

## Results

### Initial investigation of maternal cholesterol levels and CHD risk

**Fig. 1:**
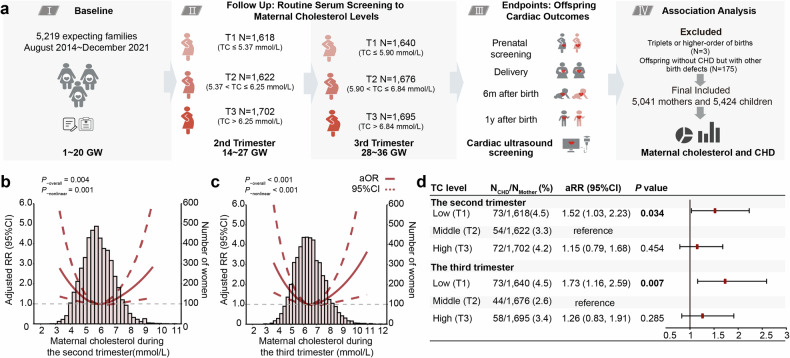
Associations between maternal cholesterol levels and incidence of CHD. **a** Scheme for the prospective cohort study to identify the association between maternal cholesterol and CHD: A total of 5041 mother‒infant pairs were eligible for the present study. Maternal cholesterol levels were assessed via serum biochemical detection at the time of card construction and delivery inspection. The cardiac defects of the infants were assessed via color sonography during prenatal screening, delivery, and at six months or one year after birth. The participants were divided into three groups according to maternal cholesterol levels by quantiles: T1 as the low cholesterol level group, T2 as the reference group, and T3 as the high cholesterol level group. **b**, **c** Dose‒response relationships of maternal TC levels during the second (**b**) and third (**c**) trimesters with the risk of CHD in offspring. The bars show the distribution of maternal TC levels. The graphs show the RRs (solid lines; 95% CIs [dotted lines]) of the associations. **d** The risk of CHD associated with cholesterol levels in the second and third trimesters is shown in the forest plot. Analyses were adjusted for maternal age, education, pre-pregnancy BMI and periconceptional folic acid or multivitamin supplements, household income, area of residence, parity, mode of conception, diabetes and hypertension during pregnancy, plurality, and gestational week at TC detection, triglyceride and fasting blood glucose levels during the second or third trimester. GW, gestational week; TC, total cholesterol; aRR, adjusted relative risk; 95% CI, 95% confidence interval

We first examined the correlation between maternal serum cholesterol levels and the risk of CHD in our birth cohort study, which included 5041 family trios (supplementary Table 1). Fetal cardiac defects were assessed via color sonography during the prenatal or postnatal periods, whereas maternal cholesterol levels were evaluated during routine antenatal clinical examinations in the second and third trimesters (Fig. 1a). Restricted cubic spline analysis revealed a clear U-shaped association between maternal cholesterol levels and the risk of cardiac defects in offspring, indicating that both low and high maternal cholesterol concentrations were associated with increased risk (*P*_nonlinear_ = 0.001 and <0.001 for the second and third trimesters, respectively) (Fig. 1b, c). When participants were categorized into tertiles, infants born to women with the lowest cholesterol levels (T1) presented a significantly greater incidence of cardiac defects in both the second trimester (RR = 1.52, *P* = 0.034, 95% CI = 1.03–2.23) and the third trimester (RR = 1.73, *P* = 0.007, 95% CI = 1.16–2.59) than did those in the reference group (T2). Although the trend in the high-cholesterol group (T3) did not reach statistical significance, the observed upward trend supports a U-shaped association (Fig. 1d).

### Impact of maternal cholesterol reduction on offspring cardiac development in a mouse model

**Fig. 2:**
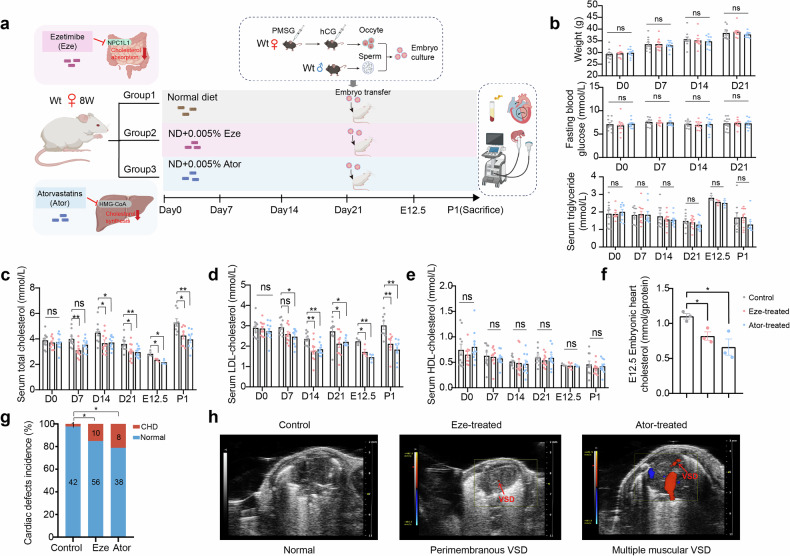
Validation of increased CHD risk in offspring from maternal low-cholesterol mouse models. **a** Schematic overview of the treatment protocol used to establish a low-cholesterol maternal mouse model. Eight-week-old (8 W) female mice were administered either ezetimibe (Eze) or atorvastatin (Ator) through their diet. The timeline encompasses mating, treatment, and critical stages of embryonic development leading to sacrifice. **b** Maternal body weight, fasting blood glucose, and serum triglyceride levels from different treatment groups at various time points (D0, D7, D14, D21, E12.5, P1) (*n* = 10 per group, except for E12.5, *n* = 3 per group). **c**–**e** Serum cholesterol levels measured at different time points. **c** Total cholesterol, **d** LDL cholesterol, and **e** HDL cholesterol (*n* = 8–10 per group, except for E12.5, *n* = 3 per group). **f** Embryonic heart cholesterol levels at E12.5 in different treatment groups (*n* = 3 per group). **g** Incidence of cardiac defects in offspring from the control, Eze-treated, and Ator-treated groups. The data are presented as percentages. The numbers marked on the bar chart represent the number of mice in each group, categorized as CHD and normal. **h** Representative images of the apical four-chamber view of hearts from the control, Eze-treated, and Ator-treated groups via echocardiography. The red blood flow indicated by the arrow corresponds to the blood flow in the interventricular septum. The data are presented as the means ± SEMs. Statistical analyses were performed via Student’s *t* test for (**b**–**f**) and Fisher’s exact test for (**g**), with *p* < 0.05 indicated by *, *p* < 0.01 indicated by **, and *p* ≥ 0.05 indicated by ns. Wt wild-type, ND normal diet, PMSG pregnant mare serum gonadotropin, hCG human chorionic gonadotropin

To further investigate the impact of maternal cholesterol reduction on offspring cardiac development, we treated 8-week-old ICR female mice with either the intestinal cholesterol absorption inhibitor ezetimibe (Eze) or the HMG-CoA reductase inhibitor atorvastatin (Ator) via their diet (Fig. 2a). Maternal body weight, fasting blood glucose, and serum triglyceride levels remained unchanged throughout the treatment period (Fig. 2b). At embryonic day 12.5 (E12.5), maternal serum cholesterol levels in the Eze- and Ator-treated groups were reduced by an average of 16.4% and 23.5%, respectively, compared with those in the control group, primarily through a decrease in low-density lipoprotein (LDL) cholesterol levels with no significant effect on high-density lipoprotein (HDL) cholesterol levels (Fig. 2c–e). These changes simulated the low cholesterol levels observed in our birth cohort.

After three weeks of treatment, wild-type C57BL/6 J two-cell embryos were transferred into treated and control ICR female mice. Maternal cholesterol reduction significantly decreased embryonic cardiac cholesterol levels at E12.5, mirroring the changes in maternal serum cholesterol (Fig. 2f). At postnatal day 1 (P1), echocardiographic analyses revealed a marked increase in the incidence of CHD in offspring from Eze- and Ator-treated mothers. The CHD rates were 2.3% (1/43) in the control group, 14.2% (10/66) in the Eze-treated group, and 17.4% (8/46) in the Ator-treated group (Fig. 2g, supplementary Table 2). The most common CHD subtype observed was ventricular septal defects (VSDs), as shown in representative echocardiography images (Fig. 2h). These findings suggest that maternal cholesterol deficiency significantly increases the risk of CHD in offspring.

### The *Cyp51*^*I383V*^ variant disrupted cholesterol synthesis and led to CHD phenotypes

**Fig. 3:**
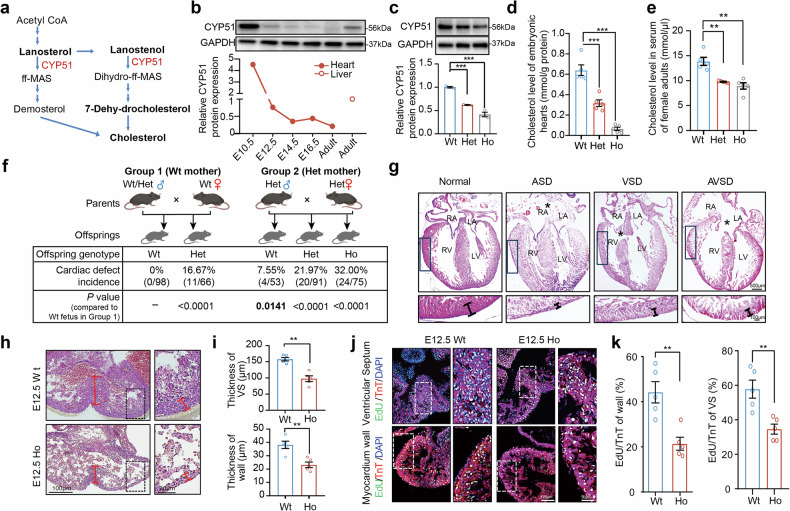
*Cyp51*^*I383V*^ impaired cholesterol synthesis, causing cardiac developmental abnormalities. **a** Schematic diagram of the cholesterol biosynthetic pathway. **b** Western blot analysis of cardiac CYP51 in wild-type embryonic and adult mouse hearts. Each sample was normalized to the total protein content, and GAPDH was used for normalization. **c** Western blot analysis and quantification of *Cyp51* levels in embryonic hearts at E12.5. **d** Quantification of cholesterol levels in embryonic hearts of Wt and *Cyp51*^*I383V*^ mutants at E12.5. **e** Quantification of cholesterol levels in the serum of female adult Wt and *Cyp51*^*I383V*^ mutants. **f** Breeding scheme and incidence of cardiac defects in the offspring of neonatal pups born to parents of different genotypes. Statistical analysis was carried out via Fisher’s test. **g** H&E staining of representative newborn *Cyp51* mutant hearts from each category. The asterisk indicates the septation defect. Note the thinning of the compactum myocardium in the insets. **h** H&E staining of embryonic hearts showing thinning of the ventricular septum and myocardial compactum. Staining was performed on four sections per sample, and a total of five samples from each genotype and treatment group were subjected to experiments. **i** Measurement of ventricular septum thickness as described in (**h**). **j** Immunofluorescence staining and **k** quantification of incorporated EdU and the cardiac myocyte marker troponin T (TnT) at the myocardium wall and ventricular septum of E12.5 Wt and *Cyp51*^*I383V*^ mutant embryonic hearts. Staining was performed on four sections per sample, and a total of five samples from each genotype and treatment group were subjected to experiments. Bars represent the means ± SEMs. Statistical analysis was performed via Student’s *t* test for (**c**–**e**) and (**i**, **k**) and the Fisher test for (**f**), with *p* < 0.05 indicated by *, *p* < 0.01 indicated by ** and *p* < 0.001 indicated by ***. Wt wild-type, Het heterozygote, Ho homozygote, VS ventricular septum

Although delayed inhibition of cholesterol synthesis or absorption can significantly increase the risk of CHD in offspring, none of the participants in our cohort survey reported exposure to these medications. To better replicate the occurrence of CHD in clinical settings, we aimed to identify functional variants in the cholesterol synthesis pathway and establish a genetic mouse model for further investigation. CYP51, previously reported as a key enzyme for cholesterol synthesis when knocked out, completely eliminates de novo cholesterol biosynthesis in *Cyp51*^*−/−*^ embryos, resulting in 100% lethality at E15 and CHD.^19^ We found that, in addition to its known expression in the adult liver, CYP51 is expressed in the developing heart (Fig. 3a, b). On the basis of these findings, we analyzed the *CYP51A1* gene in 103 CHD cases from the birth cohort to identify suitable variants for constructing a mouse model. As shown in supplementary Fig. 1a, we selected functional variants in *CYP51A1* with a minor allele frequency (MAF) of less than 0.001 in the gnomAD database across all populations and a combined annotation-dependent depletion (CADD) score >20. No loss-of-function (LoF) mutations were identified among the CHD patients. However, we identified a missense variant meeting the specified criteria, with *CYP51A1*^*I383V*^ (c.1147 A > G, p.Ile383Val) being the only recurrent functional variant observed in CHD patients, and it was maternally inherited (supplementary Fig. 1b, c). Additional genotyping in two independent Jiangsu cohorts confirmed this association. In cohort I, which included 957 atrial septum defect (ASD)/VSD patients and 2391 controls, MAF_Case_ = 0.0131 vs. MAF_Control_ = 0.0034 (OR = 3.96 [2.09–7.48], *P* = 2.29×10⁻⁵). In cohort II, which included 840 other CHD cases and 2,052 controls, MAF_Case_ = 0.0220 vs. MAF_Control_ = 0.0046 (OR = 4.93 [2.82–8.62], *P* = 2.24 × 10⁻⁸) (supplementary Fig. 1d). Although other genes (e.g., *TM7SF2*, *LBR*, and *MVD*) harbored three different rare variants in the cohort, they were not prioritized for functional studies, as they were nonrecurrent and their knockout animal models do not exhibit cardiovascular phenotypes relevant to CHD,^20–22^ precluding their suitability for mechanistic studies or knock-in model construction.

We subsequently generated a point mutation mouse model via the CRISPR/Cas9 system (supplementary Fig. 2a, b) without predicted off-target sites (supplementary Fig. 2c) to study the effects of the mutation on CHD phenotypes in vivo. Protein immunoblotting revealed a significant reduction in the number of embryos at E12.5 in the mutant fetus (Fig. 3c). The results revealed that cholesterol levels in E12.5 heart tissues were reduced in mice heterozygous for the mutant *Cyp51* allele and were almost undetectable in homozygous mutants (Fig. 3d). Moreover, female adult mice carrying the mutant *Cyp51* allele also presented decreased serum cholesterol levels (Fig. 3e).

Homozygous knockout of the *Cyp51* gene in mice causes embryonic lethality, with CHD being one of the phenotypes.^19^ However, in our study, the *Cyp51*^*I383V*^ variant segregated in the offspring according to the expected Mendelian ratio (Supplementary Table 3). Hematoxylin and eosin (H&E) staining of newborn heart sections revealed that 24 out of 75 homozygous *Cyp51*^*I383V*^ mutants presented congenital defects, representing 32.00% penetrance of the phenotype (Fig. 3f). Interestingly, heterozygous newborn pups also presented CHD phenotypes (16.67% of those born to wild-type mothers vs. 21.97% to heterozygous mothers). Moreover, even some noncarrier pups (7.55%) born to heterozygous mothers presented with heart defects (*P* = 0.0141 for *Fisher’s* exact test) (Fig. 3f). The predominant CHD subtypes were ASD (18.67%), VSD (10.67%), and atrioventricular septum defect (AVSD) (2.67%) (supplementary Fig. 3a). The compact myocardium appeared thinner in all affected hearts than in normal controls (Fig. 3g and Supplementary Fig. 3b, c). Early during development (E12.5 and E14.5), homozygous *Cyp51*^*I383V*^ mutant hearts already presented a defective ventricular septum and thinner ventricular walls (Fig. 3h, i and Supplementary Fig. 3d, e). These structural abnormalities were accompanied by reduced expression of key cardiomyocyte marker genes (i.e., *Mlc2a*, *Mlc2v*, and *Tnnt2*), whereas the expression of endothelial markers remained unchanged (supplementary Fig. 3f). Consistent with these findings, immunofluorescence staining revealed a marked reduction in cardiomyocyte proliferation (Fig. 3j, k and supplementary Fig. 3g, h) in both the ventricular septum and the myocardial wall. Taken together, these results indicate that the CHD phenotypes observed in *Cyp51*^*I383V*^ mutant hearts likely stem from impaired CM proliferation during embryonic development.

### Disrupted in situ cholesterol synthesis leads to decreased Hh signaling and GATA4 expression

**Fig. 4:**
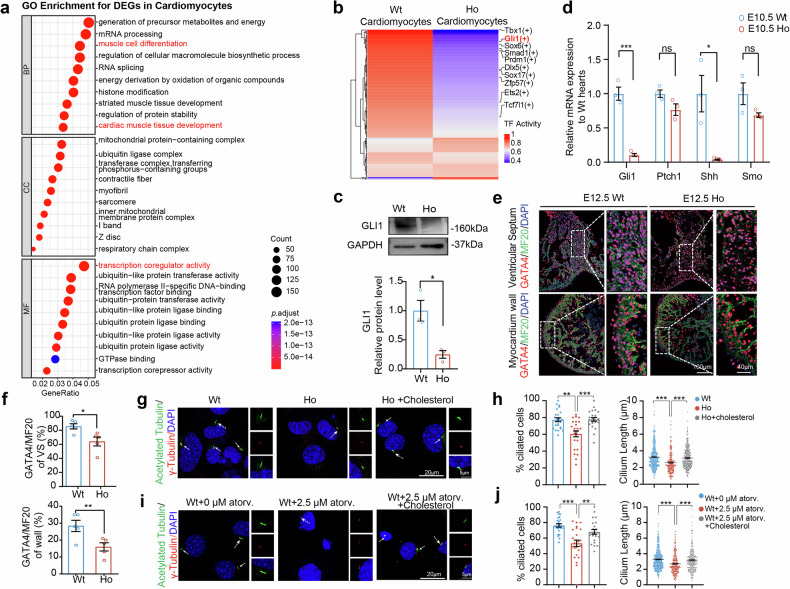
Cholesterol deficiency caused by the *Cyp51*^*I383V*^ variant led to impaired Hedgehog pathway activity and abnormal ciliogenesis. **a** Gene Ontology (GO) enrichment analyses of the scRNA-seq datasets with differentially expressed genes (DEGs) (*p*.adjust<0.05) in the cardiomyocytes of E10.5 Wt and *Cyp51*^*I383V*^ mutant hearts. The top 10 enriched biological process (BP), cellular component (CC), and molecular function (MF) pathways are displayed in bubble charts. Pathways related to cardiac development and transcriptional regulation are marked in red. **b** Heatmap showing the transcription factors with significant differences (*p*.adjust < 0.05)) in transcriptional activity between the CMs of E10.5 Wt and *Cyp51*^*I383V*^ mutants. The transcription factors annotated on the right side were the top ten significantly downregulated transcription factors known to be associated with cardiogenesis. **c** Relative protein levels of GLI1 in E10.5 Wt and *Cyp51*^*I383V*^ mutant hearts. Bars represent the means ± SD, *n* = 3. **d** Relative mRNA levels of important Hedgehog signaling pathway molecules (*Gli1*, *Ptch1*, *Shh*, *Smo*) in E10.5 Wt and *Cyp51*^*I383V*^ mutant hearts. Bars represent the means ± SD, *n* = 3. **e** Immunofluorescence staining and **f** quantification of GATA4^+^ cardiomyocyte markers at the myocardial wall and ventricular septum of E12.5 Wt and *Cyp51*^*I383V*^ mutant embryonic hearts. Staining was performed on four sections per sample, and a total of five samples from each genotype and treatment group were subjected to experiments. The statistical analysis was carried out via the unpaired two-tailed Student’s *t* test. **g** Wt and *Cyp51*^*I383V*^ mutant mouse embryonic fibroblast (MEF) cells were incubated in the presence or absence of cholesterol for 48 h and then immunostained for acetylated tubulin (green) and gamma-tubulin (red). Scale bar, 20 µm. **h** The left bar graph and right dot plot represent the quantification of the percentage of ciliated cells and cilia length in (**g**), respectively. The data are presented as the means ± SEMs (eight sections per sample, *n* = 3 samples), and 100–250 cells were scored per condition per sample via Student’s *t* test. **i** MEFs were treated with DMSO or atorvastatin in serum starvation media without or with cholesterol replenishment for 48 h and then immunostained for acetylated tubulin (green) and gamma-tubulin (red). Scale bar, 20 µm. **j** The left bar graph and right dot plot represent the quantification of the percentage of ciliated cells and cilia length in (**i**). The data represent the mean ± SEM (eight sections per sample, *n* = 3 samples), and 100–250 cells were scored per condition per sample via Student’s *t* test, with *p* < 0.05 indicated by *, *p* < 0.01 indicated by **, and *p* < 0.001 indicated by ***

To investigate the mechanism of abnormal cardiac formation in *Cyp51*^*I383V*^ mutant hearts, we performed single-cell RNA sequencing on wild-type and homozygous *Cyp51* mutant hearts at the E10.5 stage. The major clusters were subsequently annotated on the basis of their defining marker gene expression (supplementary Fig. 4a, b). Pathway enrichment analysis of differentially expressed genes (DEGs) in cardiomyocytes revealed associations with cardiac muscle tissue development, muscle cell differentiation, and transcription coregulator activity (Fig. 4a). To explore these findings further, we used SCENIC to analyze transcription factor activity in cardiomyocytes and observed a significant decrease in the activity of GLI1, a key effector of the Hedgehog signaling pathway, in mutant cardiomyocytes (Fig. 4b). Previous studies have highlighted the critical role of cholesterol in hedgehog signaling, where it acts as a posttranslational adduct by forming covalent bonds with ligands.^10,23^ As expected, we detected a significant decrease in GLI1 protein expression in mutant hearts (Fig. 4c), along with decreased mRNA expression of *Shh* and *Gli1*, confirming impaired Hedgehog signaling pathway activity (Fig. 4d).

Several studies have highlighted the essential role of Hedgehog signaling in regulating cardiac progenitor cells, specifically in maintaining the balance between CM proliferation and differentiation.^24^ To investigate this further, we analyzed the expression of specific first and second heart field marker genes.^25^ Interestingly, we found that *Gata4* expression was significantly downregulated in *Cyp51*^*I383V*^ mutant hearts (supplementary Fig. 4c). *GATA4* has been previously implicated in myocardial proliferation and interatrial development, aligning the cardiac phenotypes observed in *Cyp51*^*I383V*^ mutants.^26^ Consistently, immunostaining revealed a significant reduction in GATA4-positive cardiomyocytes in both the ventricular septum and the myocardial walls of the mutant hearts (Fig. 4e, f). To further investigate the regulatory interaction between *Gli1* and *Gata4*, we performed CUT&Tag analysis on wild-type E10.5 hearts. This analysis confirmed that *Gli1* directly binds to the promoter region of *Gata4* (supplementary Fig. 4d). Together, these results demonstrate that the inhibition of cholesterol synthesis by the *Cyp51*^*I383V*^ variant disrupts Hh signaling, further affecting its downstream target gene, *Gata4*.

The observed decrease in *Shh* mRNA expression in our study cannot be fully explained by the covalent binding of cholesterol to Hh ligands. A study by Jonathan et al. revealed that cholesterol could also bind noncovalently to vesicular transport-related proteins, including RAB11.^15^ *Rab11* has been reported to mediate vesicle trafficking to the basal body of the primary cilium, thereby playing a crucial role in ciliogenesis.^27,28^ Accordingly, we evaluated the percentage of ciliated cells and measured the length of cilia in mouse embryonic fibroblast (MEF) cells derived from the *Cyp51*^*I383V*^ mutant or treated with atorvastatin (supplementary Fig. 5a). Our findings revealed a significant reduction in both the number and length of cilia in mutant or atorvastatin-treated cells compared with those in wild-type cells. Supplementation with high-cholesterol medium restored the intracellular cholesterol levels in both the *Cyp51*^*I383V*^ mutant MEFs and the atorvastatin-treated MEFs (supplementary Fig. 5b, c). Notably, these ciliary defects were also rescued under these conditions (Fig. 4g–j). Given that abnormal ciliogenesis can lead to disturbed Hedgehog signaling^29^ and that the role of cilia in CHD pathogenesis has been highlighted by a large-scale mouse forward genetic screen,^30^ our data suggest that the inhibition of cholesterol may contribute to defective ciliogenesis and inhibited Hh signaling in developing hearts.

### Reversible pathogenic effects of a maternal high-cholesterol diet (HCD) in mice

**Fig. 5:**
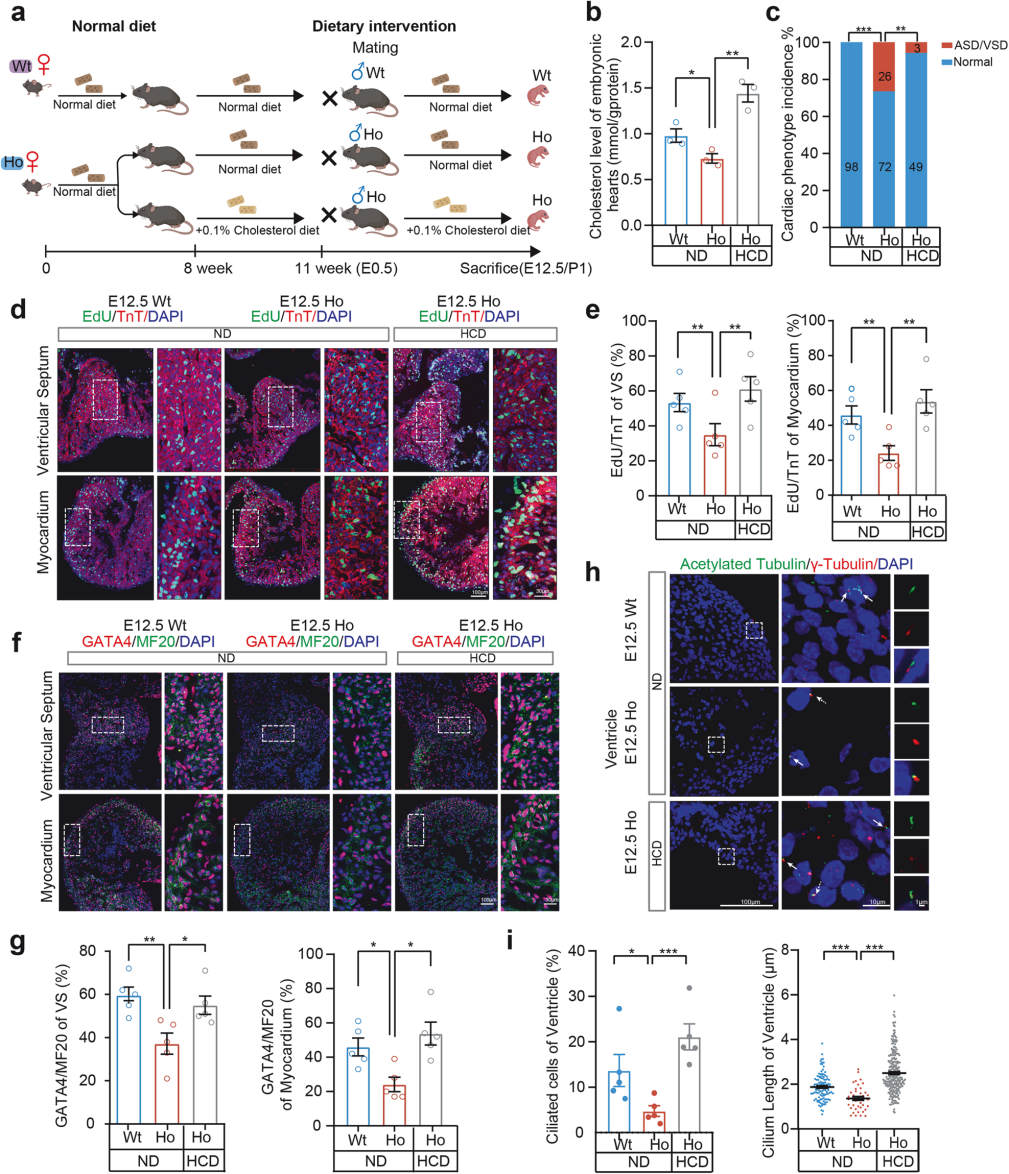
Preconditioning *Cyp51*^*I383V*^ carrier mothers with a high cholesterol diet reduces CHD risk in offspring. **a** Breeding scheme of the high-cholesterol diet (HCD) model: We preconditioned *Cyp51*^*I383V*^ mutant adult mice on normal and high-cholesterol diets for three weeks and continued the same dietary treatment for pregnant doles. H&E staining of newborn *Cyp51* mutant hearts was performed to analyze the incidence of cardiac defects in the ND and HCD groups. **b** Quantification of cholesterol levels in Wt and *Cyp51*^*I383V*^ mutant hearts in different groups at E12.5. **c** Analysis of the incidence of cardiac defects among the offspring of mothers with ND compared to those with HCD was conducted. The bar charts illustrate the quantity of newborn pups in each category. The statistical analysis was carried out via the *Fisher* test. **d** Immunofluorescence staining and **e** quantification of incorporated EdU and the cardiac myocyte marker troponin T (TnT) at the myocardium wall and ventricular septum of E12.5 ND and HCD embryonic hearts. Staining was performed on four sections per sample, and a total of five samples from each genotype and treatment group were subjected to experiments. **f** Immunofluorescence staining and **g** quantification of GATA4^+^ cardiomyocyte markers at the myocardium wall and ventricular septum (VS) of embryonic hearts at E12.5 in the ND and HCD groups. Staining was performed on four sections per sample, and a total of five samples from each genotype and treatment group were subjected to experiments. **h** Immunofluorescence staining of acetylated tubulin and gamma tubulin in the ventricles of E12.5 ND and HCD-fed embryonic hearts. **i** Quantification of the percentage of ciliated cells and cilia length in (**h**). Data represent the mean ± SEM (*n* = 5 samples), and 100–250 cells were scored per condition per sample via Student’s *t* test, with *p* < 0.05 indicated by *, *p* < 0.01 indicated by **, and *p* < 0.001 indicated by ***

Finally, we investigated whether maternal influence could be leveraged to reduce CHD risk. To this end, we preconditioned *Cyp51*^*I383V*^ mutant mice on a high-cholesterol diet for three weeks and continued the same dietary treatment throughout pregnancy (Fig. 5a). This feeding intervention increased cholesterol levels in homozygous embryonic hearts (Fig. 5b) and significantly reduced the incidence of cardiac defects in *Cyp51*^*I383V*^ mutants from 26.5% to 5.8% (Fig. 5c). Consistently, the high-cholesterol diet during pregnancy also restored cardiomyocyte growth (Fig. 5d, e), restored GATA4 expression in the ventricular septum and myocardium at E12.5 (Fig. 5f, g), and rescued ciliogenesis (Fig. 5h, i). These findings suggest that maternal dietary cholesterol plays a crucial role in offspring heart development and may serve as a potential intervention strategy for preventing CHD in generally predisposed individuals.

## Discussion

In the present study, we established a significant association between maternal cholesterol deficiency and an increased risk of CHD in offspring through both a cohort study and a mouse model. In the *Cyp51*^*I383V*^ mouse model, reduced cholesterol synthesis led to impaired Hedgehog signaling and defective ciliogenesis, ultimately resulting in cardiac abnormalities. Most notably, we identified a reversible effect by supplementing the maternal diet with high cholesterol. This finding underscores the critical role of maternal cholesterol in fetal cardiac development and suggests that appropriate maternal dietary interventions could mitigate the risk of CHD. Our results shed light on the previously underexplored influence of maternal cholesterol levels on offspring health and suggest potential interventions for reducing CHD risk through dietary modifications.

To date, very few prospective studies have comprehensively examined the environmental factors associated with CHDs.^31^ One of the most significant recent discoveries is the protective effect of periconceptional multivitamin supplements containing folic acid, which may reduce CHD risk in offspring, similar to its well-established role in preventing neural tube defects.^32,33^ However, less attention has been given to other crucial maternal factors, such as cholesterol, which is fundamental for various cellular processes critical to fetal development. Maternal cholesterol levels have long been recognized as critical factors in fetal development,^34^ given their fundamental role in maintaining cell membrane integrity, permeability, and fluidity.^35^ Moreover, cholesterol is essential for the Hh signaling pathway, which is critical for embryonic development.^9^ Our cohort study, combined with mouse models, revealed that low maternal cholesterol levels significantly increased the risk of CHD in offspring. In mouse models, inducing low cholesterol levels during pregnancy via the use of statins or ezetimibe resulted in a significant increase in CHD incidence in offspring, suggesting a causal role of maternal cholesterol deficiency in CHD pathogenesis. However, our cohort survey revealed no reported exposure to these medications.

To better mimic clinical CHD occurrence, we combined WGS analysis and identified the pathogenic *CYP51A1*^*I383V*^ variant, which we further investigated via genetic mouse models. The *CYP51A1*^*I383V*^ variant disrupts cholesterol synthesis, leading to impaired Hh signaling and ciliogenesis, both of which are essential for normal cardiac development.^36^ These findings align with those of previous studies demonstrating that mutations in key genes involved in cholesterol synthesis and Hh signaling can lead to severe developmental defects.^37,38^ The population-based variant model confirmed the damaging effects of *CYP51A1*^*I383V*^ on cholesterol synthesis and its mechanistic role in CHD pathogenesis. Importantly, our study demonstrated a reversible effect of maternal cholesterol deficiency on *CYP51A1*^*I383V*^-induced CHD risk. Supplementing the maternal diet with high cholesterol significantly reduced the incidence of cardiac defects in offspring, suggesting potential public health applications for CHD prevention.

Mechanistically, cholesterol depletion in *Cyp51*^*I383V*^ mutants was associated with shorter and fewer primary cilia, suggesting that cholesterol is required not only for the modification of Shh signaling molecules but also for correct ciliogenesis. In earlier research conducted by Jonathan and colleagues, a chemoproteomic approach was utilized that involved clickable and photoreactive sterol probes, in conjunction with quantitative mass spectrometry, to comprehensively chart cholesterol-protein interactions within living cells, revealing more than 250 proteins that bind to cholesterol.^15^ Among these, cholesterol was reported to non-covalently bind RAB11, facilitating its localization to vesicle membranes. Previous study demonstrated that Rabin8, a guanine nucleotide exchange factor (GEF) activating the Rab8 GTPase, is required for ciliary assembly. Rab8-dependent ciliary assembly is initiated by the relocalization of Rabin8 to Rab11-positive vesicles that are transported to the centrosome.^39^ However, a reduced level of cholesterol disrupted the transport of Rabin8 vesicles and blocked cilia formation.^40^ These results indicate that adequate levels of cholesterol are essential for accurate ciliogenesis, which subsequently facilitates appropriate ciliary signaling and normal development of the embryo. Given that *Gata4* is a downstream target of Hh signaling and a core regulator of cardiac transcription, its downregulation in *Cyp51*^*I383V*^ mutants likely contributes to the observed defects in septal development, including ASD and VSD.^26,41,42^

Unlike classical pathogenic variants in familial CHD, *CYP51A1*^*I383V*^ exhibited incomplete penetrance in both the population and the knock-in mouse model. Partial residual activity of *Cyp51* may allow some de novo cholesterol synthesis, as evidenced by the low but detectable cholesterol levels in mutant embryos, whereas complete knockout of *Cyp51* is embryonically lethal at E15.^19^ Environmental and maternal factors further modulate penetrance: maternal cholesterol contributes substantially to fetal cholesterol pools (approximately 60%) and can partially compensate for impaired embryonic synthesis.^34,43^ Accordingly, maternal high-cholesterol diets significantly mitigated the incidence of CHD in *Cyp51*^*I383V*^ offspring, highlighting the interplay between genetic and maternal environmental factors in modulating disease risk.

Additionally, our cohort study revealed that 1.21% of the 2,729 genotyped offspring carried loss-of-function variants in enzymes of the cholesterol-biosynthesis pathway (e.g., *DHCR7*, *SC5D*, *CYP51A1*). Of these pathogenic alleles, 41.7% were maternally inherited, 55.5% paternally inherited, and only 2.8% arose de novo, underscoring that the bulk of reproductive risk is traceable to pre-existing parental genotypes rather than new mutations. This inheritance pattern argues for routine pre-conception genetic counseling in families with dyslipidaemia or a history of CHD, followed by first-trimester maternal cholesterol screening and, when indicated, individualized dietary supplementation to ensure that fetal cholesterol supply is not limited at the critical window of cardiac septation. Interestingly, we also noted a U-shaped relationship between the levels of cholesterol in mothers and the occurrence of CHD, indicating that both low and high cholesterol levels may increase CHD risk. Thus, maintaining moderate cholesterol levels is crucial for preventing elevated CHD risk. While these observational findings provide important insights into the relationship between maternal cholesterol and CHD risk, they are derived from a prospective cohort study and cannot define the optimal maternal cholesterol range. Determining the ideal cholesterol level for preventing CHD will require further investigation through rigorously designed randomized controlled trials to establish causal relationships and provide evidence-based guidance for maternal dietary interventions.

In conclusion, by integrating prospective cohort data with a *Cyp51*^*I383V*^ mouse model, we demonstrate that maternal cholesterol insufficiency is a quantifiable, modifiable determinant of fetal cardiac development. Targeted dietary support that sustains mid-gestational cholesterol within a narrow, optimal window decreased CHD incidence in mutant pups by nearly half, indicating that population-level prevention is feasible. Future work must define the precise threshold of maternal total cholesterol that maximizes Hedgehog signal strength without provoking embryonic oxidative stress, and then translate this value into culturally acceptable, genotype-specific food-based interventions for high-risk families.

## Materials and methods

### Birth cohort study population

The study was approved by the Institutional Review Board of Nanjing Medical University (NJMUIRB [2017] 002), and informed consent was obtained from all participants or their legal guardians. This study was conducted within the Jiangsu Birth Cohort (JBC), a prospective, population-based birth cohort in Jiangsu Province, China. Women undergoing assisted reproductive technology (ART) treatment and those with spontaneous conception in the first trimester were enrolled, and both groups were followed uniformly across gestation. The details of the cohort design and data have been described elsewhere.^44^ In the present study, we examined the association between maternal cholesterol levels and the risk of CHD by including all women who had pregnancies up to 20 weeks of gestation between August 2014 and December 2021 from Nanjing Center and had at least one routine serum screening for total cholesterol levels from 20 to 36 gestational weeks (*N* = 5219). We then excluded mother–infant pairs because the mother conceived triplets or higher-order births (*N* = 3). Additionally, we excluded 175 pairs in which the mother gave birth to children without CHD but with other birth defects. Finally, 5041 mothers and 5424 children were included in the final association analysis.

#### Maternal cholesterol exposure assessment

Maternal serum total cholesterol was measured during routine second- and third-trimester obstetric examinations at the Nanjing Center via a Beckman Coulter AU5800 automated analyzer. Total cholesterol was quantified via the CHOD–PAP method with a commercial assay kit (Fosun Diagnostics, #IFU-00068). In brief, the assay involved mixing serum with reagents, incubating at 37 °C, and measuring the absorbance at 505/520 nm before and after the addition of a second reagent. The cholesterol concentration was calculated from the change in sample absorbance relative to that of a calibrator. Daily analysis of the Beckman Coulter AU system control materials ensured quality control. The participants were categorized into tertiles on the basis of maternal cholesterol levels: T1 (lower), T2 (reference), and T3 (higher).

#### Congenital heart defect outcome assessment

Health-related data, including diagnoses and ICD-10 codes, were extracted from the electronic medical records of hospitals providing antenatal care and delivery services to cohort participants, under authorized access. The cardiac defects of the infants were assessed via color sonography at four time points: prenatal, delivery, six months after birth, and one year after birth. The cases consisted of septal defects (ASD, VSD) and other types of CHD, including TOF, patent foramen ovale, and patent ductus arteriosus.

### Whole-genome sequencing (WGS) of CHD cases in the birth cohort

In the analysis of 103 CHD cases within the birth cohort, we conducted whole genome sequencing (WGS) to explore pathogenic variants related to CHD in the cholesterol metabolism pathway. Genomic DNA from both the offspring and their parents was extracted from peripheral blood samples and sequenced with the Illumina HiSeq X Ten platform. The library preparation adhered to the manufacturer’s guidelines (Illumina). Briefly, the extracted DNA was fragmented, size-selected through gel electrophoresis, and subsequently ligated to oligonucleotide adapters to form paired-end libraries. These libraries underwent amplification via ligation-mediated PCR using adapter-specific primers to ensure their enrichment. Sequencing generated 150 bp paired-end reads, aimed at an average coverage of approximately 30× for each sample. The processes of alignment, variant calling, quality control (QC), and variant annotation were executed as outlined in prior research.^45^ To pinpoint functional missense variants, we evaluated the frequency of rare variants (MAF < 0.001 as per the gnomAD database) and deleterious variants (with a combined annotation-dependent depletion score exceeding 20) among the 103 CHD cases in our birth cohort.

### CHD case‒control genotyping study population

All experiments involving these human subjects were approved by the Ethics Committee of Nanjing Medical University (No. 410, 2024), and written informed consent was obtained from all participants. A total of 1797 CHD cases were recruited from two independent cohorts at affiliated hospitals between 2006 and 2012, with diagnoses confirmed by echocardiography or surgery. Patients with syndromic anomalies, chromosomal abnormalities, positive family history of CHD, or relevant maternal exposures were excluded, and 2 ml of whole blood was collected from all participants for DNA extraction. Non-CHD outpatient controls were recruited during the same period, excluding those with congenital anomalies or chronic heart disease.

For Cohort Ⅰ, we selected 957 nonsyndromic CHD cases (including ASD, VSD and combined ASD/VSD) and 2391 non-CHD outpatients as described above, a portion of which were also included in our previous GWAS on common genetic variants.^46^ For Cohort II, 840 other subtype cases (including tetralogy of Fallot, partial/complete atrioventricular canal, pulmonary stenosis, coarctation of the aorta, partial/total anomalous pulmonary venous drainage, and patent ductus arteriosus) and an additional 2052 controls were recruited from the First Affiliated Hospital and the Affiliated Nanjing Children’s Hospital of Nanjing Medical University.

### Genotyping and quality control of CHD cases and controls

We genotyped two cohorts of 1797 nonsyndromic CHD cases and 4443 controls via the TaqMan allelic discrimination assay on the ABI 7900 system (Applied Biosystems, Foster City, CA, USA). The detailed information regarding the primers and probes is shown in Supplementary Table 4. Genotyping quality was controlled using multiple strategies: (i) case and control samples were randomized on each plate; (ii) technicians performing genotyping were blinded to CHD status; (iii) each 384-well plate included two blank controls; and (iv) four samples validated by Sanger sequencing served as positive internal standards, ensuring at least two heterozygotes per plate.

### Animal husbandry

All animal procedures were conducted following the animal care guidelines approved by the Nanjing Medical University Animal Care Unit (approval No. 1912049). ICR and C57BL/6 J mice were obtained from Vital River (Charles River) and the Jiangsu Laboratory Animal Center of Nanjing Medical University (Nanjing, China), and were housed under a 12-h light/12-h dark cycle (lights off from 20:00 to 08:00).

### Construction of maternal low-cholesterol mouse models using ezetimibe and atorvastatin

To investigate the effects of maternal cholesterol reduction on offspring cardiac development, we treated 8-week-old female ICR mice with either ezetimibe (Eze) or atorvastatin (Ator) by incorporating these cholesterol-lowering agents into their diet. Specifically, the mice were fed mouse maintenance diets containing 0.005% (w/w) atorvastatin (MCE, HY-B0589) or 0.005% (w/w) ezetimibe (MCE, HY-17376), which were synthesized by Jiangsu Xietong Bioengineering Co., Ltd. The diets were changed every two days to ensure the efficacy of the treatment. Throughout the treatment period, the mice were weighed weekly, and fasting blood glucose levels were monitored via glucose test strips. Additionally, blood samples were collected from 8 to 10 mice per group, allowed to clot at room temperature, and then centrifuged at 3000 rpm for 10 min to obtain serum for the analyses of total cholesterol and other biochemical parameters.

After three weeks of treatment, wild-type C57BL/6 J embryos were transferred into treated and control ICR female mice. Briefly, four-week-old female B6 mice received intraperitoneal injections of 7IU PMSG (0.1 mL, Ningbo Second Hormone Factory [NSHF], Cat# G024) followed by 7IU HCG (0.1 mL, NSHF, Cat# GN026) followed by 7IU HCG (0.1 mL) 44–48 h later. IVF was performed 13–15 h post-HCG administration. Epididymal sperm from 10-week-old male B6 mice were capacitated in TYH droplets (TYH [Yihe Biotechnology, Cat# M2030] supplemented with glutathione [Sigma‒Aldrich, Cat# G6013], preequilibrated at 37 °C for 20 min) at 37 °C for 1 h. Oviducts of superovulated female mice were placed in HTF droplets (HTF [Yihe Biotechnology, Cat# M1130] supplemented with glutathione, preequilibrated at 37 °C for 20 min). Isolated cumulus‒oocyte complexes (COCs) were co-incubated with sperm under 5% CO2 at 37 °C. After 4–6 h, the oocytes were washed in M2 medium (Sigma‒Aldrich, Cat# M7167), and zygotes exhibiting two pronuclei were selected for culture in CZB medium (Yihe Biotechnology, Cat# M1650) to the two-cell stage (37 °C, 5% CO_2_, approximately 12 h). Treated and control ICR female mice were cohoused with 10-week-old vasectomized ICR male mice at a 2:1 ratio. Vaginal plug-positive individuals were selected 12 hours post-mating to serve as embryo recipients. Sixteen graded two-cell embryos were surgically transferred into the unilateral oviductal ampulla. Post transfer, the mice were individually housed under SPF conditions. The diets were maintained until the offspring of the pregnant mice were sacrificed at either E12.5 or postnatal day 1 (P1) for further analysis.

On postnatal day 1, echocardiographic assessments (VisualSonics, Vevo 2100) were conducted in a single-blinded manner by an experienced cardiologist to screen for congenital structural heart defects in the offspring.

### Biochemical analysis of total cholesterol, triglyceride, low-density lipoprotein cholesterol (LDL-C) and high-density lipoprotein cholesterol (HDL-C)

The levels of total cholesterol, triglycerides, low-density lipoprotein cholesterol (LDL-C), and high-density lipoprotein cholesterol (HDL-C) were assessed via specific assay kits from Nanjing Jiancheng Bioengineering Institute. Total cholesterol levels in embryonic hearts, adult sera, and mouse embryonic fibroblast (MEF) cells were determined via a total cholesterol assay kit (A111-1-1). The cholesterol content in the cell lysates was measured via a colorimetric assay and expressed as mmol of cholesterol per mg of cellular protein (mmol cholesterol/mg protein). Embryonic hearts at various stages were pooled and homogenized in saline solution, followed by centrifugation. The resulting lysates were added to a 96-well plate with cholesterol reagent, incubated, and then analyzed for optical density at 510 nm. Adult serum cholesterol levels were expressed as mmol of cholesterol per liter of serum. Triglyceride levels in adult serum were determined via a triglyceride assay kit (A110-1-1) via a similar procedure. LDL-C and HDL-C in adult serum were assessed via an LDL-C assay kit (A113-1-1) and an HDL-C assay kit (A112-1-1), respectively. Serum samples were added to a 96-well plate, followed by the addition of specific reagents and incubation before the optical density was measured at 600 nm. This comprehensive biochemical analysis provided detailed insights into the lipid profiles across different sample types.

### Construction of the *Cyp51a1*^*I383V*^ mouse model

CRISPR/Cas9-mediated homology-directed repair (HDR) was used to introduce the I383V variant of *CYP51A1* into the mouse germline genome. The guide RNA sequence was derived from chr5:4087800-4087819 (GRCm38/mm10) and designed via CRISPR Design (http://crispr.mit.edu/). A DNA oligo corresponding to this sequence was subsequently cloned and inserted into PGL3 for guide RNA in vitro transcription. A mixture of Cas9 mRNA, sgRNA, and a single-stranded oligodeoxyribonucleotide (ssODNA) HDR template carrying the I383V variant was microinjected into single-cell C57BL/6 mouse embryos. The edited sites were amplified by PCR and Sanger sequencing in the founder mice. We successfully generated C57BL/6 founder mice carrying the I383V variant, which were subsequently backcrossed for at least four consecutive generations with congenic C57BL/6 mice to establish an isogenic background. Potential off-target sites were amplified via PCR and analyzed via Sanger sequencing in two F1 heterozygotes, but no off-target mutations were found. Additionally, progeny from all C57BL/6 × *CYP51A1*^*I383V*^ backcrosses segregated according to the expected Mendelian ratio at weaning (Supplementary Table 3). The primer sequences and their respective amplicon sizes used for genotyping each mutant mouse line are listed in Supplementary Table 4.

### RNA isolation and qRT-PCR

Total RNA was extracted via TRIzol Reagent (Invitrogen) and reverse transcribed via the High-Capacity RNA-to-cDNA Kit (Takara). Approximately 1000 ng of RNA was used for reverse transcription with PrimeScriptTM RT Master Mix (Takara, Japan). Using SYBR Premix Ex Taq™ (Takara), the cDNA was amplified in triplicate on a QuantStudio™ 7 Flex Real-Time PCR System. The primer sequences and their respective amplicon sizes used for genotyping are provided in Supplementary Table 4.

### Western blotting

Tissues and cells from embryonic mouse hearts (E10.5/E12.5) were gathered and lysed using the universal protein extraction lysis buffer RIPA (Beyotime, P0013B) that includes a cocktail of protease inhibitors (MedChemExpress, HY-K0010). The protein lysates were centrifuged, and the resulting supernatants were quantified via a BCA protein assay kit (Beyotime, P0012). The lysates were subsequently denatured at 100 °C for 10 min with 5× SDS‒PAGE sample buffer (Beyotime, P0015L). For western blotting, 40 μg of protein was separated on 8% or 10% SurePAGE™ gels, depending on the molecular weight of the protein (GenScript, M00661/M00666), and then placed onto a polyvinylidene difluoride (PVDF) membrane (Millipore, ISEQ00010). The antibodies utilized for western blotting included the following: anti-CYP51A1 (Abcam, ab210792, 1:1000 dilution), anti-GLI1 (Cell Signaling Technology, 2534, 1:1000 dilution), and anti-GAPDH (Cell Signaling Technology, 5174, 1:2000 dilution).

### Histological analysis of the heart

The hearts of newborn fetuses and fetuses at E12.5 and E14.5, respectively, were dissected and fixed in 4% paraformaldehyde (PFA) for 24 hours. The hearts were then sectioned at 8 μm intervals and stained with H&E. The slides were read and analyzed blindly without revealing the genotype of the sample.

### Immunofluorescence staining

Embryo hearts were collected, washed with PBS, and then fixed in 4% PFA for two hours on ice. After being washed with PBS, the hearts were dehydrated in 30% sucrose overnight at 4 °C. The tissues were then embedded in optimal cutting temperature compound (Sakura, 4583) and stored at −80 °C. Cryosections of 15 µm thickness were collected from the positively charged slides. For immunofluorescence staining, the tissues were first washed three times with PBS, followed by blocking with goat serum for 30 min at room temperature. Primary antibodies were incubated overnight at 4 °C. After washing with PBS, secondary antibodies were added along with DAPI (Alexa Fluor 488; Alexa Fluor 546) for 2 h at room temperature. The antibodies used were as follows: GATA4 (Abcam, ab84593; 1:400 dilution), cTnT (Abcam, ab8295; 1:100 dilution), MF20 (Developmental Studies Hybridoma Bank; 1:100 dilution), Ki67 (GB1303030-2, 1:200 dilution), γ-tubulin (Sigma-Aldrich, T6557, 1:1000 dilution), and acetyl-α-tubulin (Cell Signaling, D20G3, 1:1000 dilution). Images were processed via ImageJ software and analyzed in a blinded manner with respect to the genotype and treatment groups.

### Single-cell RNA sequencing

E10.5 wild-type and homozygous *CYP51A1*^*I383V*^ mouse embryos were isolated and dissected to obtain hearts. The hearts were washed 2–3 times with DPBS precooled at 4 °C and then transferred to a new 1.5 mL centrifuge tube containing collagenase II. The cell dissociation solution was transferred to a C tube containing enzymatic cell dissociation solution, and a gentleMACS Dissociator (Miltenyi Biotec) was used to digest the tissue. The resulting suspension was filtered through a 70- or 40-µm cell strainer and centrifuged at 4 °C. After the supernatant was removed, the cell pellet was resuspended in 1 mL of cell resuspension solution. Subsequently, 3 mL of red blood cell lysis buffer was added, and the sample was incubated on ice for 2–10 min. To stop lysis, 6 mL of DMEM containing 5% weight/volume FBS was added. Finally, after centrifugation at 4 °C, the supernatant was discarded, and the pellet was resuspended in 1 mL of the abovementioned mixture for the first subsequent cell quality inspection. If any additional processing steps were performed, cell quality inspection was conducted again. After being stained with AO/PI double fluorescent dye, the cell quality was assessed via a Countstar Rigel S2 to determine the cell concentration, viability and clumping ability. At the same time, Trypan blue staining was performed to evaluate cell morphology, cell concentration, and the presence of cell fragments and impurities.

For quality inspection, the concentration of single-cell suspensions was adjusted to 700–1500 cells/μL, and single-cell capture and library construction were performed according to the instructions of the Chromium Next GEM Chip G Single Cell Kit (10× Genomics, PN-1000120) and the Chromium Next GEM Single Cell 3ʹ GEM (Library & Gel Bead Kit v3.1, 10× Genomics, PN-1000121), respectively. For the single-cell suspensions, single-cell separation and cDNA synthesis were performed via a Chromium Controller (10× Genomics, GCG-SR-1). Next, cDNA was recovered via magnetic beads, followed by amplification and purification. cDNA quality inspection was subsequently conducted via a Qubit 4.0% & dsDNA HS Assay (Thermo, Q32854) and an Agilent 4150 & High Sensitivity D5000 Tape station to determine the cDNA concentration and integrity, respectively. The 3′ gene expression library was constructed from the quality-qualified cDNA via the Chromium Next GEM Single-Cell 3′ GEM protocol. The library was quantified following fragmentation, adapter ligation, and sample index PCR. The pooled libraries were then sequenced on an Illumina NovaSeq 6000 platform to generate 150-bp paired-end reads.

### Single-cell RNA sequencing analysis

Raw FASTQ files were demultiplexed and aligned to the mouse reference genome (mm10) via the Cell Ranger (*v.6.1.1*) pipeline with default settings. Cell filtering, data normalization, and unsupervised analysis were carried out via the R package Seurat (*v4.1.0*) following its recommended steps.^47^ In brief, genes expressed in fewer than 10 cells and cells meeting any of the following criteria were filtered out: mitochondrial genes >20%, unique molecular identifiers (<500 or >50,000), and gene count (<500 or >6000). Doublets were detected and removed via the R package DoubletFinder (*v2.0.3*). The remaining data were then normalized and scaled via the SCT method. A total of 30 principal components (PCs) were selected based on the Seurat *ElbowPlot* and *JackStrawPlot* functions. To mitigate technical batch effects, Seurat integration was applied. Cell clustering was performed using the *FindClusters* function (algorithm = Leiden), and t-distributed stochastic neighbor embedding (tSNE) visualization was generated via *RunTSNE*. Marker genes for each cluster were identified with the *FindAllMarkers* function using default settings, and the identities of the clusters were annotated based on the expression levels of established marker genes.

Differential gene expression analysis within cell types between Wt and Ho was performed via the *FindMarkers* function in Seurat, which utilizes a likelihood ratio test. Genes with *p*.adjust < 0.05 were considered differentially expressed. Gene Ontology (GO) expression analysis was performed via the clusterProfiler R package v4.2.2.

In order to examine the activity of transcription factors (TFs) within individual cells, SCENIC analysis was performed using pySCENIC (version 0.12.0). The necessary databases for this analysis included the TF database (mm10 10 kbp upstream and 10 kbp downstream full transcription clustered, specifically the genes_vs_motifs.rankings.feather) as well as the motif annotation database (mgi.v10.m0.001), both of which were sourced from the pySCENIC website (https://github.com/aertslab/pySCENIC). The input for pySCENIC comprised the raw count matrix produced by Seurat, and the quantification of TF activity was determined by calculating the area under the recovery curve (AUC) for genes influenced by each transcription factor. To assess which TFs were differentially activated between Wt and Ho cardiomyocytes, statistical significance was evaluated using the Wilcoxon rank sum test (with adjustments for *p*-values set at <0.05). The findings were represented through heatmap visualizations.

### Cell culture

Immortalized mouse embryonic fibroblasts (MEFs) (FuHeng Biology, Shanghai, China) were cultured in high-glucose Dulbecco’s modified Eagle’s medium (DMEM) (Gibco, C11965500BT) supplemented with 10% fetal bovine serum (Gibco, 10099141 C), 100 IU/ml penicillin, and 100 µg/ml streptomycin at 37 °C in a humidified atmosphere containing 5% CO_2_. Primary MEFs were isolated from E13.5 embryos. Briefly, embryos were minced via a sterile razor blade, followed by the addition of 4 ml of 0.25% trypsin-EDTA (Gibco, 25200-056). The samples were incubated at 37 °C for 15 min. Following the incubation period, the tissue fragments were placed into 10 cm tissue culture dishes, subjected to multiple pipetting to dissociate the cells, and then incubated overnight in DMEM/F12 medium (Gibco, C11330500BT) with the addition of 10% fetal bovine serum. It was confirmed that all cell lines were free from mycoplasma contamination.

On the day prior to the experiment, MEFs were introduced into 24-well plates or 10-cm dishes. The next day, the culture medium was switched from a complete formulation to a starvation medium (DMEM or DMEM/F12 with 0.1% serum and 1% Pen/Strep), enriched with atorvastatin (MedChemExpress, HY-B0589) or DMSO, and maintained for 48 h.

### CUT & Tag

CUT&Tag (Cleavage Under Targets and Tagment) experiments and high-throughput sequencing were performed by SeqHealth Technology Co., Ltd. (Wuhan, China). Two sample groups, each comprising 10 fresh E10.5 hearts from wild-type mouse embryos, were homogenized to isolate cells, which were then bound to Concanavalin A-coated magnetic beads. Cells were incubated with an anti-GLI1 antibody (R&D Systems, AF3324) for 2 h, followed by a 1-h incubation with a secondary antibody at room temperature. Hyperactive pG-Tn5 transposase was subsequently applied for tagmentation. The reaction was stopped, and DNA fragments were extracted using PCI, then amplified by PCR with indexed P5 and P7 primers. The resulting libraries were enriched, quantified, and sequenced on an Illumina NovaSeq 6000 platform with 150-bp paired-end reads.

Raw sequencing reads were processed using Fastp (v0.23.1) to remove low-quality reads and trim adapter sequences. Clean reads were aligned to the mouse genome (GRCm38/mm10) with Bowtie2 (v2.2.6) using default settings. Sambamba (v0.7.1) was employed for SAM/BAM format conversion and removal of PCR duplicates. Read distribution around transcription start sites (TSS) was visualized with DeepTools (v2.4.1). Peak calling was performed with MACS2 (v2.2.7.1), and peak annotation and distribution analyses were carried out using BEDTools (v2.30.0).

### Cholesterol replenishment in vitro

In the rescue experiments, the highest concentration of water-soluble cholesterol (Sigma-Aldrich, C4951) that maintained cell viability was utilized. Prior to the starvation process, homozygous *Cyp51*^*I383V*^ and control MEFs underwent a pretreatment with 4 mM cholesterol for 1 h. Following washing with PBS, the cells were subjected to starvation for a duration of 48 h. Due to the high toxicity of additional cholesterol to control cells, a modified protocol was used for MEFs treated with atorvastatin: 2.5 µM atorvastatin was added at the start of starvation, and after 24 h, 4 mM cholesterol was co-administered for 1 h. Starvation and atorvastatin treatment were then continued for another 24 h prior to fixation or lysis.

### Dietary high cholesterol rescue

A group of 8-week-old Ho female mice was preconditioned on a high-cholesterol diet for three weeks. High-cholesterol (HCD) feed (0.1% cholesterol, 18% lard, and 81.9% basic feed) was purchased from Jiangsu Xietong Bioengineering Co., Ltd. Two other groups of 8-week-old Wt and Ho female mice were fed a normal diet as controls. The diets of the three groups were maintained until the offspring of the pregnant mice were sacrificed at either E12.5 or P1.

### Statistical analysis

To assess the associations between maternal cholesterol levels (second and third trimesters) and offspring CHD risk, we investigated potential nonlinear associations via restricted cubic spline (RCS) models, analyzing maternal total cholesterol (mmol/L) as a continuous variable separately for each trimester. We further performed multivariable logistic regression to determine risk ratios (RRs) and 95% CIs. The genotyped CHD case‒control association analysis was performed via an additive logistic regression model.

For in vitro and in vivo experiments, the data are presented as the means ± SEMs. Statistical analyses were performed via two-tailed Student’s *t* tests for parametric data and Fisher tests for categorical data. *P < 0.05, **P < 0.01, ***P < 0.001.

## Supplementary information

Supplemental materials

Original and uncropped films of Western blots

## Data Availability

The data from scRNA-seq have been submitted to the Gene Expression Omnibus (GEO) and can be found under the accession number GSE307566. All other source data supporting the findings of this study, including experimental datasets, CUT&Tag data, and mouse echocardiography videos, have been deposited in figshare (10.6084/m9.figshare.30204643).
