# Supplementary material for: Maternal cholesterol deficiency predisposes congenital heart defects risk
Source: Signal Transduct Target Ther. 2025 Nov 12;10:366. doi: 10.1038/s41392-025-02463-w (PMC12606117; doi:10.1038/s41392-025-02463-w)
Supplement: Supplementary file 1 — Supplemental materials [file 41392_2025_2463_MOESM1_ESM.docx]

Supplementary Materials for

Maternal cholesterol deficiency predisposes congenital heart defect risk

Yayun Gu^1,2,3#^, Jimiao Gao^1,2#^, Hong Lv^1,2#^, Yan Zhou^1,2#^, Tao Jiang^1,2#^, Jia Guo^1,2^, Wanting Ma^1,2^, Yiwei Cheng^1,2^, Xia Chi^4^, Qi Xi^5^, Kan Ye^6^, Jiangbo Du^1,2^, Jiong Li^1,2^, Cheng Wang^1,2^, Juncheng Dai^1,2^, Hongxia Ma^1,2^, Guangfu Jin^1,2^, Yuan Lin^1,2^, Hongbing Shen^1,2^, and Zhibin Hu^1,2,3*^

Correspondence to: zhibin_hu@njmu.edu.cn

**This PDF file includes:**

Figures. S1 to S5

Tables S1 to S4

**Other Supplementary Materials for this manuscript include the following:**

Original and uncropped films of Western blots


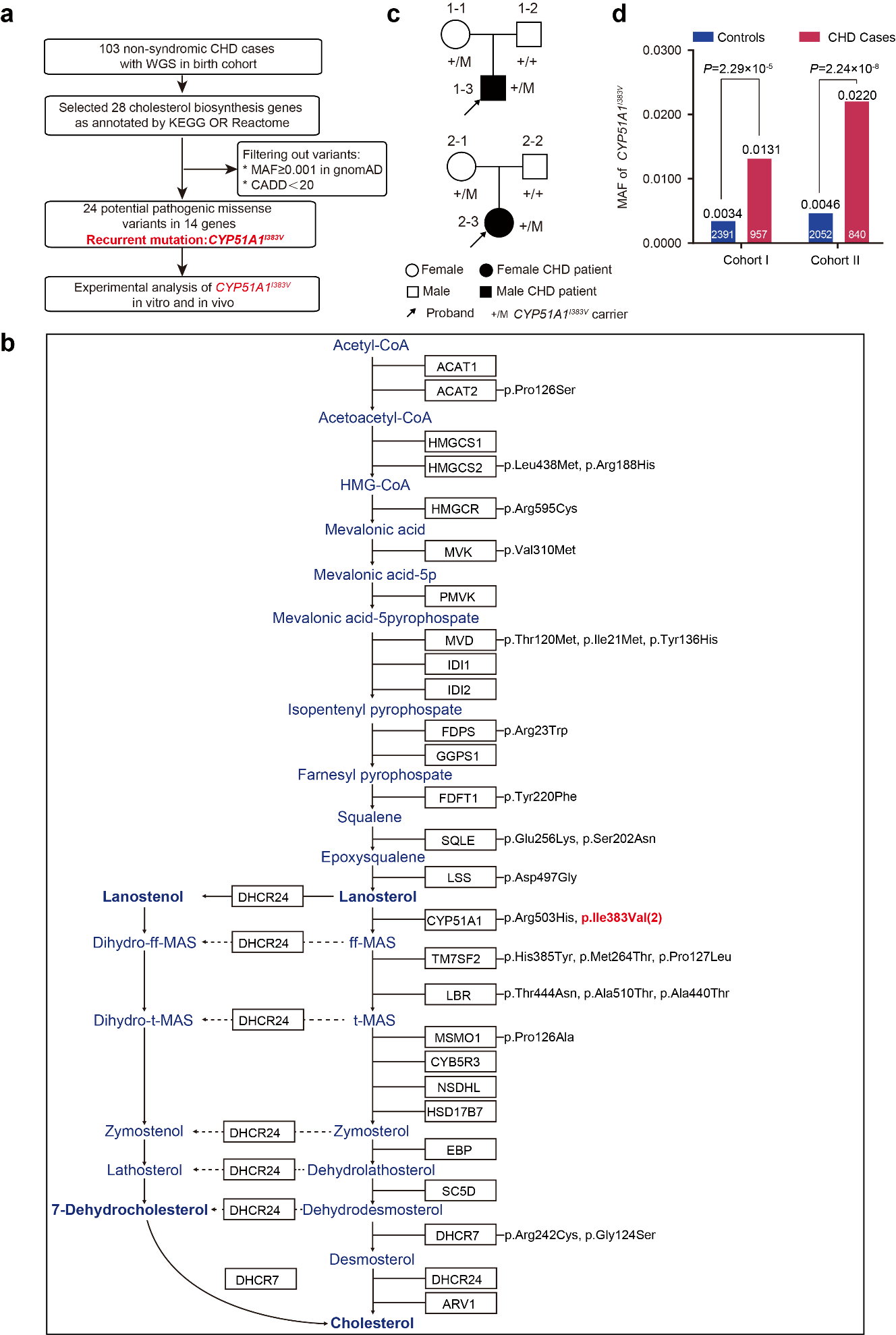


Figure. S1.

**Functional variants of genes involved in cholesterol synthesis pathway in 103 non-syndromic CHD offspring in our birth cohort population with target sequencing data.** **Related to Figure 1. a,** Flowchart of the screening process for functional variants in *CYP51A1* in 103 non-syndromic CHD offspring with whole genome sequencing data in our birth cohort. **b,** Flowchart showed all the genes, enzymes, and functional missense variants on the genes in cholesterol synthesis pathway. **c,** Pedigree charts of two families from our birth cohort, showing the inheritance of the *CYP51A1* p.Ile383Val variant in CHD-affected offspring. Circles (females), squares (males), filled symbols (CHD patients), open symbols (unaffected). Proband marked with an arrow. Genotypes: +/+ (wild-type), +/M (heterozygous carrier). **d,** Comparison of MAF values of *CYP51A1^I383V^* between CHD cases and the controls in two independent cohorts. The number of case or control subjects is given at the bottom of each bar graph.


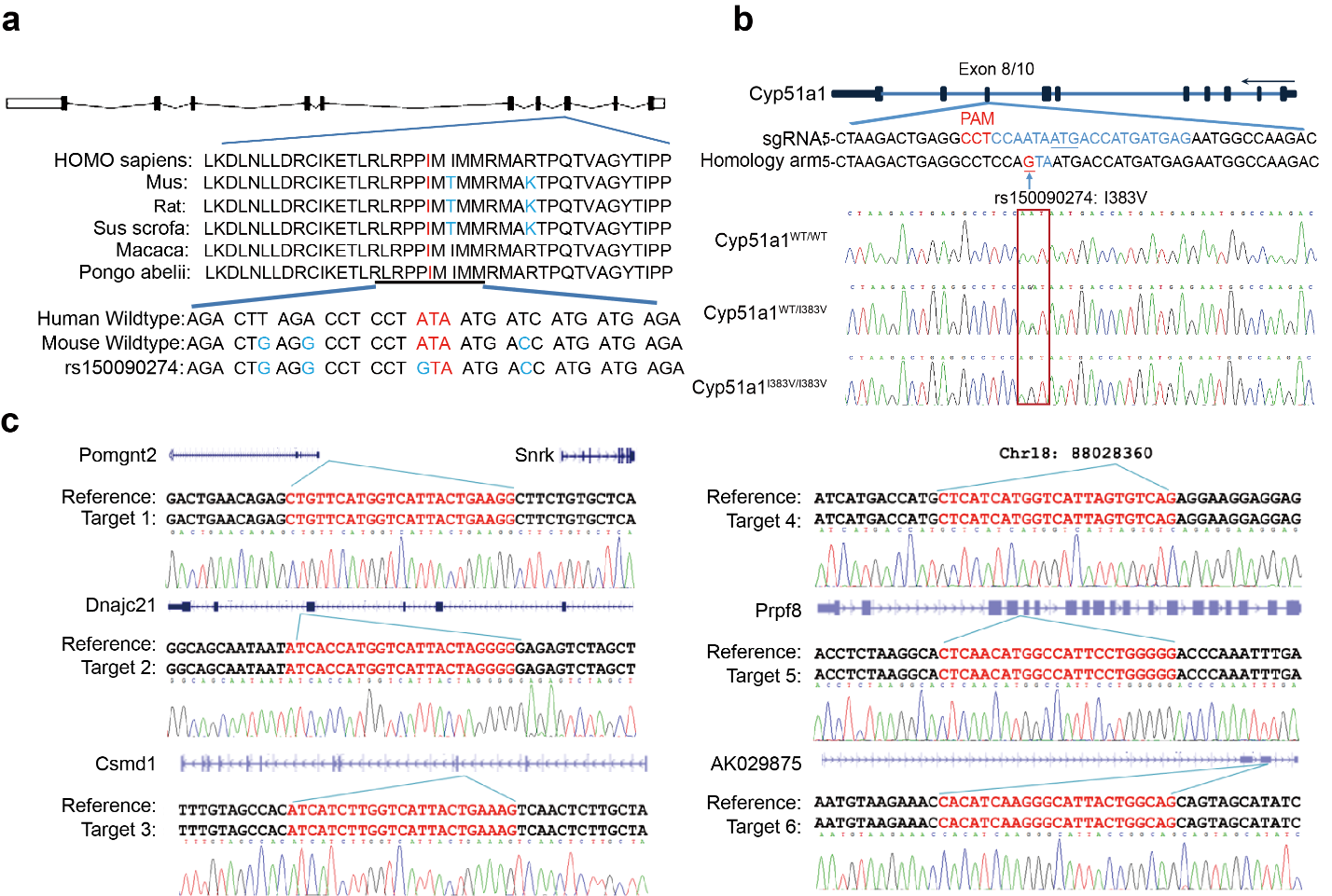


Figure. S2.

**Construction of *CYP51^I383V^* knock-in mouse. Related to Figure 3.**  **a,** The DNA and amino acid sequences were conserved between humans and mice. **b,** Schematic diagram of sgRNA targeting on *Cyp51*. PAM sequence was highlighted in red, while targeting sits are in blue. The ssODNA HDR template contains an A to G variant. Sanger sequence of genomic DNA shows the ATA in WT VS (A/G) TA in heterozygote and GTA in homozygote. **c,** The predicted 6 off-target sites of sgRNA were amplified by PCR and sequenced among 4 heterozygotes and 4 homozygotes respectively.


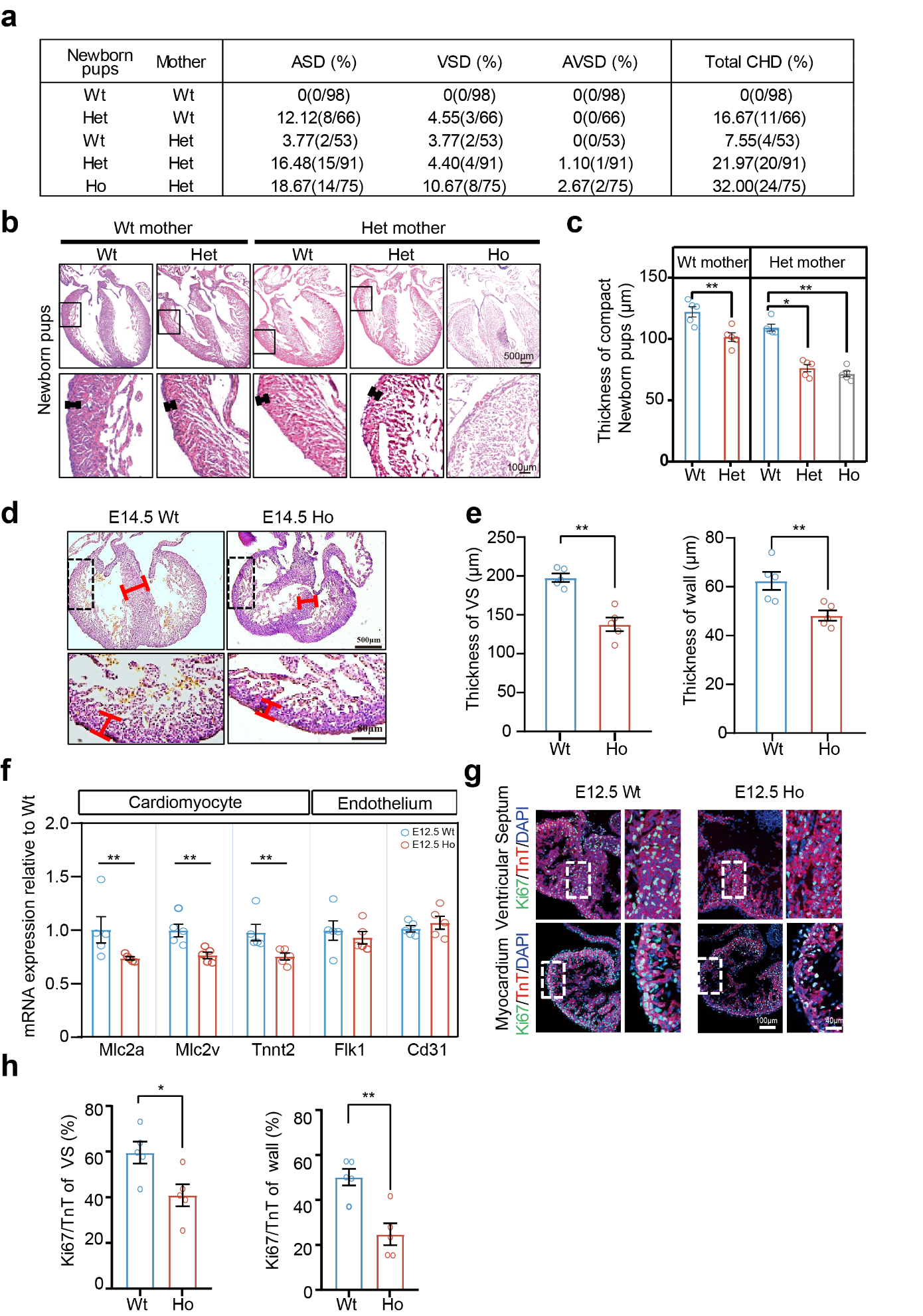


Figure. S3.

**Incidence of various CHD phenotypes in newborn pups. Related to Fig.3.** **a,** Incidence of various CHD phenotypes (ASD, VSD and AVSD) in newborn pups. **b,** H&E staining of hearts of newborn pups were used to assess the thickness of myocardial compact. The staining was performed on four sections per sample, and total five samples of each genotype and treatment group were experimented. **c,** Quantitative thickness of myocardial compact in newborn pups as in (c), n=5 and the bars represent mean ± SEM. The statistical analysis was carried out using unpaired two-tailed Student’s t-test. **d,** H&E stained sections from E14.5 hearts to assess the thickness of ventricular septum and wall in Wt and mutant hearts. The staining was performed on four sections per sample, and total five samples of each genotype and treatment group were experimented. **e,** Quantitative thickness of myocardial compact in embryonic hearts at E14.5, n=5 and the bars represent mean ± SEM. The statistical analysis was carried out using unpaired two-tailed Student’s t-test. **f,** The expression of cardiomyocyte and endothelial marker genes were assessed in embryonic hearts of E12.5, n=4 and the bars represent mean ± SEM. The statistical analysis was carried out using unpaired two-tailed Student’s t-test. **g,** Immunofluorescence staining and (**h**) quantification thereof of Ki67^+^ cardiomyocyte at myocardium wall and ventricular septum of E12.5 control and *Cyp51^I383V^* mutant embryonic hearts, n=5 and the bars represent mean ± SEM. The statistical analysis was carried out using unpaired two-tailed Student’s t-test.


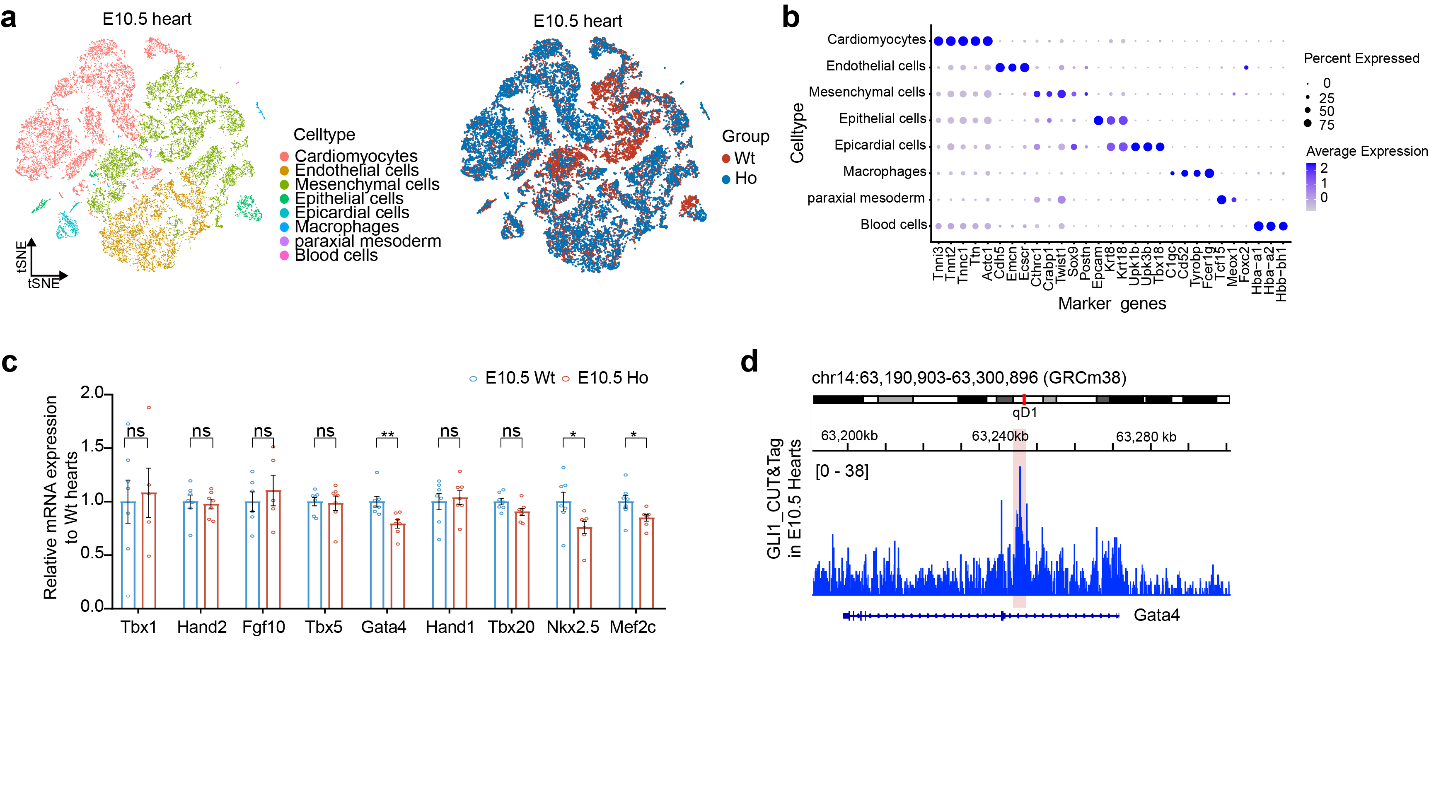


Figure. S4.

**Single-cell RNA sequencing revealed Hedgehog regulation of target gene *Gata4*. Related to Fig.4. a,** tSNE plot of 8 main cell types (left) and integrated datasets (right) of Wt and *Cyp51^I383V^* mutants E10.5 heart single-cell RNA-Seq. **b,** Dot plot of cell type marker genes. Cell specific marker genes were selected according to previous study. The color of dots represents average expression, and size of dots represents average percent of cells expressing selected gene. **c,** mRNA expression analysis referred to GAPDH in embryonic hearts of E12.5 to verify marker genes specifically expressed in the first and second heart fields. n=7 and the bars represent mean ± SEM. The statistical analysis was carried out using unpaired two-tailed Student’s t-test. **d,** Integrative Genomics Viewer (IGV) tracks showing the location of *Gli1* at *Gata4* locus from CUT&Tag analysis in Wt E10.5 mouse hearts.


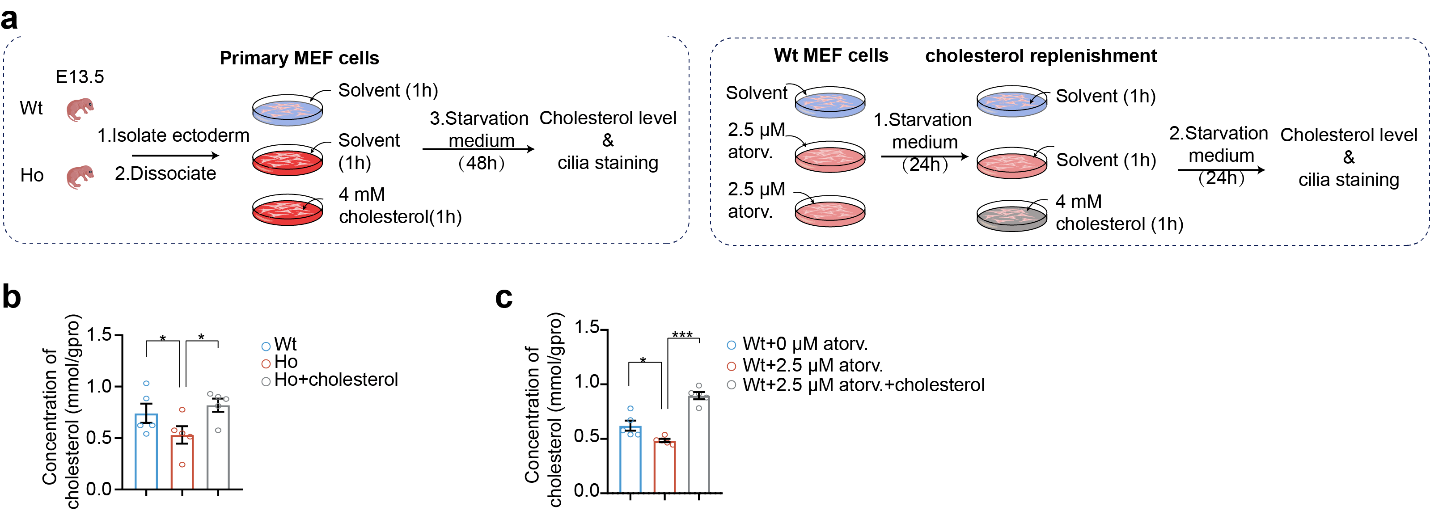


Figure. S5.

**Role of cholesterol in ciliogenesis. Related to Fig.4.** **a,** Schematic of experimental procedures for assessing cholesterol's impact on cilia formation in MEF cells. Primary MEF cells are treated with solvent and cholesterol to evaluate cilia formation. Wt MEF cells are treated with atorvastatin to deplete cholesterol, followed by cholesterol replenishment to restore cilia. **b,** The concentration of cholesterol after 48 hours serum-starvation in wild-type and mutant MEF cells without or with cholesterol replenishment. **c,** The concentration of cholesterol after 48 hours serum-starvation in atorvastatin (atorv.) treated MEF cells without or with cholesterol replenishment. Data represent mean ± SEM (n = 5 experiments), Student’s t-test.

Table S1.

**Maternal and neonatal characteristics of 5,424 children of 5,041 mothers included in the prospective cohort study. Related to Fig. 1.**

| **Characteristics, No. (%)** | **Group** | **Study population** |
| --- | --- | --- |
| **Maternal characteristics** |  |  |
| **Total number of women** |  | 5041 |
| **Age, year, mean (SD)** |  | 30.09 (3.83) |
| **Age** |  |  |
|  | <25 | 316 (6.3) |
|  | 25-29 | 2483 (49.3) |
|  | 30-34 | 1656 (32.9) |
|  | ≥35 | 586 (11.6) |
| **Education, year** |  |  |
|  | <12 | 661 (13.1) |
|  | ≥12 | 4375 (86.8) |
| **Pre-pregnancy BMI, kg/m^2^, mean (SD)** |  | 21.61 (3.05) |
| **Pre-pregnancy BMI** |  |  |
|  | <18.5 | 624 (12.4) |
|  | 18.5-23.9 | 3459 (68.6) |
|  | 24-27.9 | 751 (14.9) |
|  | ≥28 | 193 (3.8) |
| **Periconceptional tobacco use** |  | 66 (1.3) |
| **Alcohol intake during pregnancy** |  | 87 (1.7) |
| **Periconceptional folic acid or multivitamin supplements** |  | 4410 (87.5) |
| **Household income, CNY** |  |  |
|  | <50,000 | 250 (5.0) |
|  | 50,000-100,000 | 1122 (22.3) |
|  | 100,000-200,000 | 2119 (42.0) |
|  | ≥200,000 | 1467 (29.1) |
| **Area of residence** |  |  |
|  | Rural | 522 (10.4) |
|  | Urban/sub-urban | 4518 (89.6) |
| **Parity** |  |  |
|  | Nulliparous | 3935 (78.1) |
|  | Multiparous | 1080 (21.4) |
| **Mode of conception** |  |  |
|  | ART conception | 1210 (24.0) |
|  | Spontaneous conception | 3831 (76.0) |
| **Diseases during pregnancy** |  |  |
|  | Diabetes | 1401 (27.8) |
|  | Hypertension | 331 (6.6) |
| **TG level during the second trimester, mmol/L, mean (SD)** |  | 2.20 (0.93) |
| **TG level during the third trimester, mmol/L, mean (SD)** |  | 2.97 (1.33) |
| **FBG level during the second trimester, mmol/L, mean (SD)**  **（Reference: 3.10-5.10）** |  | 4.54 (0.48) |
| **FBG level during the third trimester, mmol/L, mean (SD)**  **（Reference: 3.10-5.10）** |  | 4.54 (0.47) |
| **Plurality** |  |  |
|  | Singleton | 4658 (92.4) |
|  | Twins | 383 (7.6) |
| **Mode of delivery** |  |  |
|  | Vaginal | 2508 (49.8) |
|  | Cesarean | 2435 (48.3) |
| **Gestational age** **at delivery, week, mean (SD)** |  | 38.54 (2.28) |
| **Gestational age at delivery** |  |  |
|  | ≥37 | 4561 (90.5) |
|  | <37 | 480 (9.5) |
| **Neonatal characteristics** |  |  |
| **Total number of neonates** |  | 5424 |
| **Pregnancy outcome** |  |  |
|  | Liveborn | 5345 (98.5) |
|  | Stillborn | 64 (1.2) |
|  | Terminations of pregnancy | 15 (0.3) |
| **Child's sex** |  |  |
|  | Male | 2836 (52.3) |
|  | Female | 2533 (46.7) |
| **Birth weight, g, mean (SD)** |  | 3247.27 (543.40) |
| **Birth weight** |  |  |
|  | ≥2500 | 4894 (90.2) |
|  | <2500 | 421 (7.8) |

Abbreviations: BMI, body mass index; CNY, China yuan; SD, standard deviation; TG, triglyceride; FBG, Fasting blood glucose. Missing data less than 5% for education, Pre-pregnancy BMI, household income, area of residence, parity, mode of delivery, child’s sex and birth weight

Table S2.

**Frequency analysis of cardiac defects in control, Eze-treated, and Ator-treated offspring of P1 mice. Related to Fig.2.**

| Phenotype | Control (N=43) | Eze-treated (N=66) | Ator-treated (N=46) |
| --- | --- | --- | --- |
| Muscular VSD | 1 (2.3%) | 3 (4.5%) | 8(17.4%) |
| Perimembranous VSD | 0 | 2 (3.1%) | 0 |
| PDA | 0 | 5(7.6%) | 0 |

Abbreviations: VSD, ventricular septal defects; PDA, patent ductus arteriosus.

Table S3.

**Observed and expected distributions of *Cyp51^I383V^* and wildtype Pups. Related to Fig.3*.***

| Background | *Cyp51^I383V^* genotype | Number of mice | Homozygous | | Heterozygous | | Wildtype | | *P^a^* |
| --- | --- | --- | --- | --- | --- | --- | --- | --- | --- |
|  |  |  | Observed | Expected | Observed | Expected | Observed | Expected |  |
| C57BL/6J | Het-Het | 173 | 27.70% | 25% | 45.66% | 50% | 26.59% | 25% | 0.51 |
| C57BL/6J | Wt-Het | 68 | - | - | 45.59% | 50% | 54.41% | 50% | 0.47 |
| C57BL/6J | Het-Het | 247 | 27.13% | 25% | 46.56% | 50% | 26.32% | 25% | 0.55 |

^a^ χ^2^ analysis was used to evaluate the distribution of *Cyp51^I383V^* and Wt pups.

Table S4.

**Primers involved in TaqMan and PCR reactions by PCR System (Applied Biosystems).**

| **Gene name** | **Primer sequence(5'-3')** | **Tm** |
| --- | --- | --- |
| rs150090274  human genotype | 5'-GCTTTCTCATTTTAGCTCAAGGATCT | 61.0 |
|  | 3'-TGGCCATTCTCATCATGATCA |  |
|  | P-G:FAM-ATTAAGACTTAGACCTCCTGTA-MGB |  |
|  | P-A:HEX-ATTAAGACTTAGACCTCCTATA-MGB |  |
| rs150090274  mouse genotype | 5'-GAGACTATTCCCTCCTGCTGTTGAG | 62.7 |
|  | 3'-AACTGGGCTCCAGAAGCAGA |  |
| Cyp51-expression | 5'-GAGGCAACTTGCTTTCCACG | 61.0 |
|  | 3'-GTGGTGGACTTTTCGCTCCA |  |
| Cyp51-offtarget1 | 5'-CAGGATCACCTCTTCGGCTC | 61.0 |
|  | 3'-TCAAGTCCGAGCTCCAACAC |  |
| Cyp51-offtarget2 | 5'-AAGTCGTCAGCTCACATCCG | 64.8 |
|  | 3'-AGCACCAGGTTCAGTGGAAG |  |
| Cyp51-offtarget3 | 5'-GGGCCCCTCAAGGAAATGAA | 64.8 |
|  | 3'-ACCCCGGGTCTTGAGTAGAG |  |
| Cyp51-offtarget4 | 5'-TGCAGTATGAATGCAGCAAGC | 61.0 |
|  | 3'-AGTCATGTCAGCAGTGGCTAT |  |
| Cyp51-offtarget5 | 5'-CTGGGTTATCGAGCCGGTTT | 61.0 |
|  | 3'-CATGCTGCAAGCGTCTTACC |  |
| Cyp51-offtarget6 | 5'-GCAACTACTGTGGTCCACCT | 61.0 |
|  | 3'-TGTTCTGACACTGCTTGGAGC |  |
| Isl1 | 5'-ATCGAGTGTTTCCGCTGTGT | 61.0 |
|  | 3'-GCCCGTCATCTCCACTAGTT |  |
| Tbx1 | 5'-CGCTGTGGGACGAGTTCAAT | 61.0 |
|  | 3'-AGTGTACTCGGCCAGGTGTA |  |
| Hand2 | 5'-AGCTACATCGCCTACCTCATG | 61.0 |
|  | 3'-TCACTGCTCTCCTCTTCTTCAC |  |
| Fgf10 | 5'-TGTCCTGGAGATAACATCAGTGG  3'-TTTGGATCGTCATGGGGAGG | 61.0 |
| Tbx5 | 5'-ATCCCCAGCACAAACTCCAG | 61.0 |
|  | 3'-ACTCTCAGGTTTCGAATCGCA |  |
| Gata4 | 5'-ACTACCTGTGCAATGCCTGT | 61.0 |
|  | 3'-TTTGAATCCCCTCCTTCCGC |  |
| Hand1 | 5'-GCTACCAGTTACATCGCCTACT | 61.0 |
|  | 3'-CCTGGTCTCACTGGTTTAGCT |  |
| Tbx20 | 5'-GGGAAGCTGGACTGTGGTTT | 61.0 |
|  | 3'-AGAGAGGATGAGGATGGGCT |  |
| Nkx2.5 | 5'-CCCACGCCTTTCTCAGTCAA | 61.0 |
|  | 3'-CTGTCGCTTGCACTTGTAGC |  |
| Mef2c | 5'-CTCACCTGGTAACCTGAACAAGA | 61.0 |
|  | 3'-TGTTGAAGCCAGACAGAGATGA |  |
| Mlc2a | 5'-CACAACGTGGCTCTTCTAATGTC | 61.0 |
|  | 3'-GGTGTCAGCGCAAACAGTT |  |
| Mlc2v | 5'-AGACACCATGGCACCAAAGAA | 61.0 |
|  | 3'-GGGAAAGGCTGCGAACATCT |  |
| Tnnt2 | 5'-AGAAGGACCTGAATGAGCTACAG | 61.0 |
|  | 3'-GGCACAGCTTTGACGAGAAC |  |
| Cd31 | 5'-ATGGGAGGTGATGAATGGGC | 61.0 |
|  | 3'-GGCAGCGAAACACTAACACG |  |
| Flk1 | 5'-TCATCGCCTCTGTCAGTGAC | 61.0 |
|  | 3'-TGCCTCACAGAAGACCATGC |  |
| Gli1 | 5'-TGAGGTTGGGATGAAGAAGCA | 61.0 |
|  | 3'-GCATCAGAAAGGGGCGAGAT |  |
| Shh | 5'-GGGAAGATCACAAGAAACTCCGA | 61.0 |
|  | 3'-ACTGCTCGACCCTCATAGTGT |  |
| Ptch1 | 5'-AAGGGGCAAAGCTACAGTCC | 61.0 |
|  | 3'-CCCTGTCTTCATTCCAGTTGATG |  |
| Smo | 5'-CTCCTACTTCCATTCGCTCAC | 61.0 |
|  | 3'-CAAAACAAATCCCACTCACAGA |  |
| GAPDH | 5'-TTCACCACCATGGAGAAGGC | 61.0 |
|  | 3'-GGCATGGACTGTGGTCATGAG |  |
