# Supplementary figures and images for: Maternal cholesterol deficiency predisposes congenital heart defects risk
Source: Signal Transduct Target Ther. 2025 Nov 12;10:366. doi: 10.1038/s41392-025-02463-w (PMC12606117; doi:10.1038/s41392-025-02463-w)

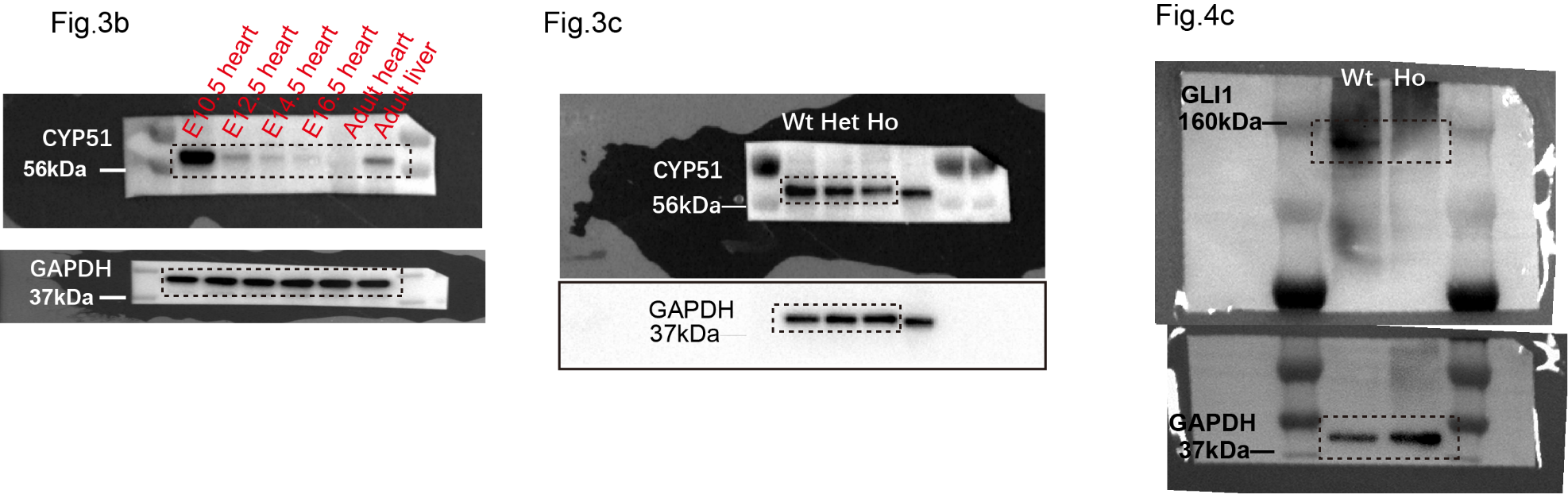

Supplement: Supplementary file 2 — Original and uncropped films of Western blots [file 41392_2025_2463_MOESM2_ESM.docx]
